# *IFT74* variants cause skeletal ciliopathy and motile cilia defects in mice and humans

**DOI:** 10.1371/journal.pgen.1010796

**Published:** 2023-06-14

**Authors:** Zeineb Bakey, Oscar A. Cabrera, Julia Hoefele, Dinu Antony, Kaman Wu, Michael W. Stuck, Dimitra Micha, Thibaut Eguether, Abigail O. Smith, Nicole N. van der Wel, Matias Wagner, Lara Strittmatter, Philip L. Beales, Julie A. Jonassen, Isabelle Thiffault, Maxime Cadieux-Dion, Laura Boyes, Saba Sharif, Beyhan Tüysüz, Desiree Dunstheimer, Hans W. M. Niessen, William Devine, Cecilia W. Lo, Hannah M. Mitchison, Miriam Schmidts, Gregory J. Pazour

**Affiliations:** 1 Center for Pediatrics and Adolescent Medicine, University Hospital Freiburg, Freiburg University Faculty of Medicine, Freiburg, Germany; 2 Human Genetics Department, Radboud University Medical Center Nijmegen and Radboud Institute for Molecular Life Sciences (RIMLS), Nijmegen, The Netherlands; 3 Program in Molecular Medicine, University of Massachusetts Chan Medical School, Biotech II, Worcester, Massachusetts, United States of America; 4 Institute for Human Genetics, Technical University Munich (TUM), School of Medicine, Munich, Germany; 5 Department of Human Genetics, Amsterdam Movement Sciences, Amsterdam UMC, Amsterdam, The Netherlands; 6 Electron microscopy Center Amsterdam, Department of Medical Biology, VUMC, Amsterdam, The Netherlands; 7 Electron Microscopy Core, University of Massachusetts Chan Medical School, Worcester, Massachusetts, United States of America; 8 Genetics and Genomic Medicine Programme, University College London, UCL Great Ormond Street Institute of Child Health, London, United Kingdom; 9 Department of Microbiology and Physiological Systems, University of Massachusetts Chan Medical School, Worcester, Massachusetts, United States of America; 10 Genomic Medicine Center, Children’s Mercy Hospital, Kansas City, Missouri, United States of America; 11 West Midlands Genomic Medicine Hub, Birmingham Women’s Hospital, Birmingham, United Kingdom; 12 Department of Pediatrics, Division of Pediatric Genetics, Cerrahpasa Medical Faculty, University-Cerrahpasa, Istanbul, Turkey; 13 Center for Pediatrics and Adolescent Medicine, University Hospital Augsburg, Augsburg, Germany; 14 Department of Pathology, Amsterdam University Medical Center (AUMC), Amsterdam, The Netherlands; 15 Department of Developmental Biology, University of Pittsburgh, 8111 Rangos Research Center, Pittsburgh, Pennsylvania, United States of America; 16 CIBSS—Center for Integrative Biological Signaling Studies, University of Freiburg, Freiburg, Germany; Washington University School of Medicine, UNITED STATES

## Abstract

Motile and non-motile cilia play critical roles in mammalian development and health. These organelles are composed of a 1000 or more unique proteins, but their assembly depends entirely on proteins synthesized in the cell body and transported into the cilium by intraflagellar transport (IFT). In mammals, malfunction of non-motile cilia due to IFT dysfunction results in complex developmental phenotypes that affect most organs. In contrast, disruption of motile cilia function causes subfertility, disruption of the left-right body axis, and recurrent airway infections with progressive lung damage. In this work, we characterize allele specific phenotypes resulting from IFT74 dysfunction in human and mice. We identified two families carrying a deletion encompassing IFT74 exon 2, the first coding exon, resulting in a protein lacking the first 40 amino acids and two individuals carrying biallelic splice site mutations. Homozygous exon 2 deletion cases presented a ciliary chondrodysplasia with narrow thorax and progressive growth retardation along with a mucociliary clearance disorder phenotype with severely shorted cilia. Splice site variants resulted in a lethal skeletal chondrodysplasia phenotype. In mice, removal of the first 40 amino acids likewise results in a motile cilia phenotype but with little effect on primary cilia structure. Mice carrying this allele are born alive but are growth restricted and developed hydrocephaly in the first month of life. In contrast, a strong, likely null, allele of *Ift74* in mouse completely blocks ciliary assembly and causes severe heart defects and midgestational lethality. *In vitro* studies suggest that the first 40 amino acids of IFT74 are dispensable for binding of other IFT subunits but are important for tubulin binding. Higher demands on tubulin transport in motile cilia compared to primary cilia resulting from increased mechanical stress and repair needs could account for the motile cilia phenotype observed in human and mice.

## Author summary

The intraflagellar transport (IFT) proteins are highly conserved across eukaryotes suggesting that each subunit has unique functions in maintaining particle structure or in carrying specific cargos from the cell body into the cilium. In this work, we explore the function of IFT74 through the study of mouse and human alleles. Previous structural work indicates that IFT74 forms a heterodimer with IFT81 and together, their N-termini form a tubulin binding domain that is thought to facilitate most tubulin transport into cilia. We identified mouse and human alleles that remove the first 40 amino acids of the IFT74 tubulin binding domain. In *in vitro* assays, the deletion did not affect the ability of IFT74 to bind IFT81 but reduced the ability of IFT74 to bind microtubules. Structurally, the mutation strongly affected motile cilia, perhaps due to a greater demand for tubulin transport into motile cilia. However, humans and mice showed phenotypes associated with both motile and non-motile ciliopathies.

## Introduction

Cilia are evolutionarily conserved, microtubule-based organelles that serve diverse sensory and motility functions throughout the eukaryotic kingdom. In mammals and other vertebrates, most cell types display non motile primary cilia. These cilia are thought to sense the extracellular milieu allowing cells to coordinate with their environment, which is critical during development and adult tissue homeostasis [1,2]. In addition, certain specialized cells display motile cilia that can move fluids over their surface and or power their movement though liquids. These cilia play critical roles in maintaining lung health and fertility, and also are critical in the establishment of the left-right pattern of the heart and other organs.

With the diversity of ciliary structure and function throughout the body, it follows that disease-causing variants (likely pathogenic and pathogenic variants) in different ciliary genes can cause strikingly different phenotypes. Typically, dysfunction of primary cilia causes complex developmental phenotypes affecting multiple organs. The organ involvement varies but commonly renal, liver and pancreatic cysts are observed along with retinal degeneration, brain abnormalities and skeletal malformations including polydactyly, brachydactyly, shortened long bones and short ribs [3,4]. In humans and mice, motile cilia dysfunction results in infertility, hydrocephaly, primary ciliary dyskinesia (PCD) / mucociliary clearance disorders and disturbances of the left-right body axis. Male infertility is thought to result from impaired sperm motility while hydrocephaly is thought to result from impaired movement of cerebrospinal fluid over the ependymal cells of the brain. PCD is characterized by reduced mucociliary clearance of the airways causing recurrent respiratory tract infections, ultimately leading to irreversible pulmonary dysfunction. Disrupted left-right patterning can result in internal organ reversal (situs inversus) or randomization (heterotaxy) [5–7]. Situs inversus is a relatively benign condition whereas heterotaxy often causes complex structural heart disease [5,8–14].

As in most eukaryotes, mammalian ciliary assembly is driven by intraflagellar transport (IFT) [15,16]. During IFT, kinesin-2 and dynein-2 motors transport large multimeric IFT trains from the cell body into and along the ciliary microtubules in the anterograde and retrograde directions respectively. The IFT trains are composed of three evolutionarily conserved subcomplexes, IFT-A, IFT-B and the BBSome. IFT-A contains 6 proteins (IFT144, IFT140, IFT139, IFT122, IFT121, and IFT43), IFT-B contains 16 proteins (IFT172, IFT88, IFT81, IFT80, IFT74, IFT70, IFT56, IFT54, IFT57, IFT52, IFT46, IFT38, IFT27, IFT25, IFT22, IFT20) and the BBSome contains eight proteins (BBS1, BBS2, BBS4, BBS5, BBS7, BBS8, BBS9 and BBIP1). While much remains to be understood about cargo binding, studies have indicated that IFT-A working with Tulp3 is important for the delivery of membrane proteins to cilia [17], IFT46 working with ODA16 delivers outer dynein arm components [18,19] and IFT74/IFT81 deliver tubulin to the organelle [20,21].

Disease-causing variants in at least five IFT-A, six IFT-B and five dynein-2 genes cause congenital syndromes with skeletal, renal, and retinal defects [22,23]. These are clinically grouped as ciliary chondrodysplasias but vary in severity. Short-rib polydactyly (SRPS) is at the severe end of the spectrum and is incompatible with survival beyond the neonatal period [24]. Jeune asphyxiating thoracic dystrophy (ATD) presentation is variable ranging from cardiorespiratory lethality due to lung hypoplasia in ~20% of cases to milder skeletal phenotypes depending on the underlying genetic cause. Interestingly, hypomorphic IFT-gene dysfunction in ATD or Cranioectodermal dysplasia (CED) causes frequent extraskeletal renal, hepatic and retinal disease in contrast to hypomorphic dynein-2 complex variants causing a severe phenotype restricted to the skeleton [25]. The pulmonary hypoplasia is thought to be a consequence of lung growth restriction *in utero* caused by the abnormally small thoracic cavity due to the shortened ribs; however, lung-patterning defects may contribute. Respiratory distress is shared with mucociliary clearance disorders, including Primary Ciliary Dyskinesia (PCD) but mucociliary clearance disorders are not usually associated with skeletal, kidney or retinal defects. Respiratory failure caused by motile ciliopathies develops slowly over several decades due to recurrent respiratory infections. Currently more than 60 mucociliary clearance disorder disease genes have been identified. These genes encode subunits of the motility apparatus, their assembly factors, or genes involved in multiciliogenesis but in general are not needed for assembly of non-motile primary cilia [26,27].

Here we report human genetic variants in the gene encoding the IFT74 subunit of IFT-B. In four independent families, the variant in *IFT74* caused an ATD/SRPS-like skeletal dysplasia spectrum phenotype. Two of these families carry a deletion of exon 2 and present with mucociliary clearance phenotypes in addition to ATD-like skeletal dysplasia features. The other two families carry splicing alleles associated with a SRPS phenotype with congenital heart defects. Mouse models showed that a complete loss of *Ift74* prevented ciliary assembly and caused severe cardiac malformations with complete lethality at about embryonic day 9. A mouse allele similar to the human exon 2 deletion formed primary cilia but had defects in the assembly of motile cilia. These mice survived gestation and were born alive but showed growth restriction in the postnatal period and developed hydrocephaly.

## Results

### Identification of individuals carrying biallelic *IFT74* loss of function variants

**Fig 1:**
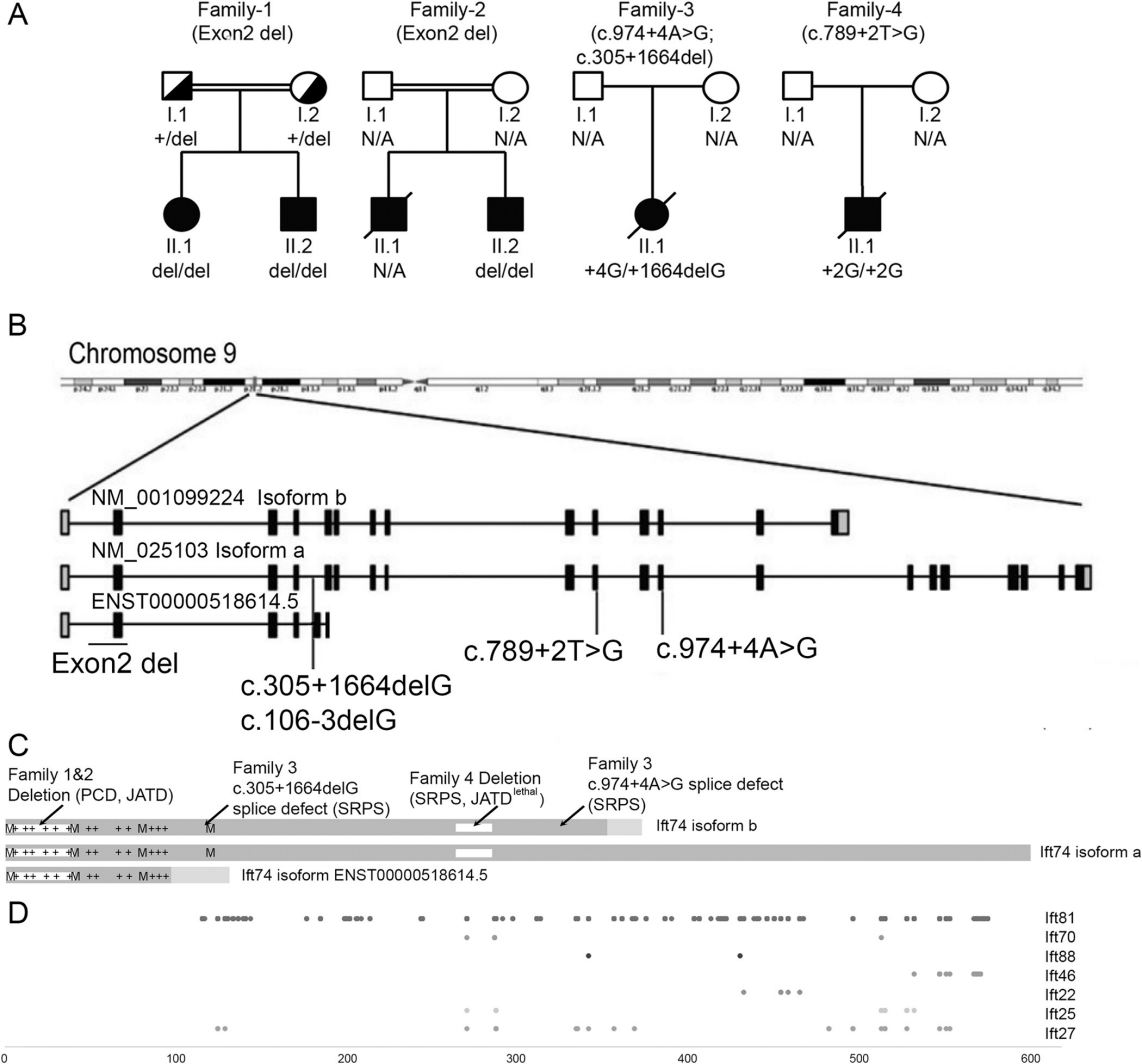
IFT74 Patients Develop Ciliopathy Phenotypes. (A) Family trees of affected individuals. (B) Diagram of the IFT74 locus showing the positions of NM_025103 (Isoform a), NM_001099224 (Isoform b), and ENST00000518614.5 transcripts. Black boxes denote coding exons. Grey boxes are 5’and 3’ non-coding exons. (C) Diagram of IFT74 protein structure. Isoform a is composed of 600 residues, isoform b 372 residues and ENST00000518614.5 146 residues. The N-terminal 351 residues of isoform b are identical to isoform a, and there are 21 unique residues at the C-terminal end. ENST00000518614.5 shares 102 residues with isoforms a and b, and has 44 unique residues at its C-terminal end. The basic, tubulin binding domain at the N-terminus, is marked with +’s to show positions of lysines and arginines. The first four methionines (M) at positions 1, 41, 80, and 121 are shown (mouse Ift74 also has a methionine at position 84). The locations of the deletions of 1–40 in family 1and 2, and 263–274 in family 4 are shown. (D) Crosslinks to IFT74 by other IFT-B proteins are shown at the bottom. These were identified in *Chlamydomonas* IFT74 [40] and mapped to corresponding positions in human IFT74 isoform a.

**Fig 2:**
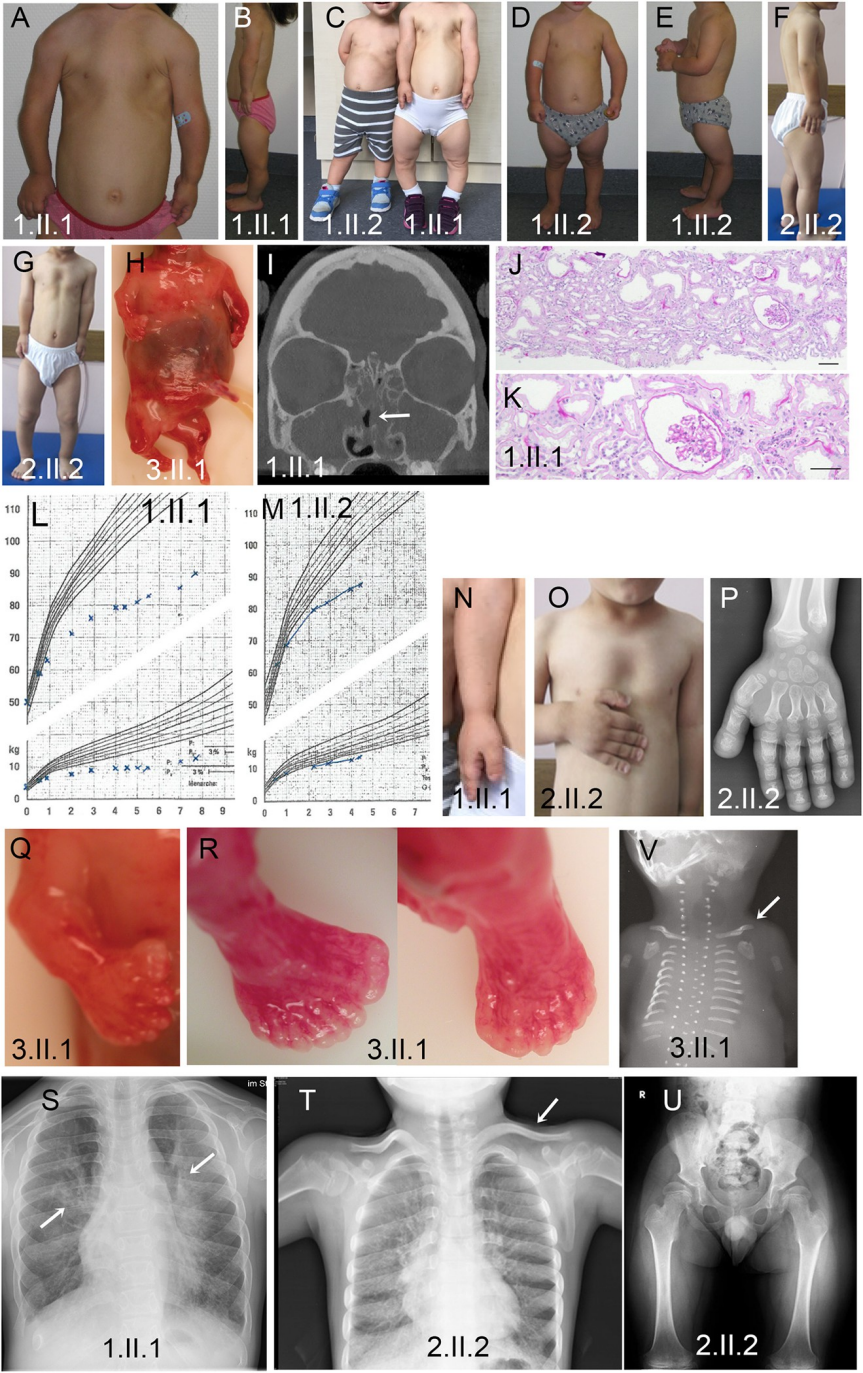
Clinical Phenotypes In IFT74 Patients. (A, C, G) Narrow thorax and shortened upper extremities in all three affected individuals, most pronounced in 1.II.1. (B-G) Shortened lower extremities in all three individuals with pronounced bowing of the lower legs. (H) Severely shorted extremities and narrow thorax in fetus 3.II.1. (I) Head CT image of patient 1.II.1 showing a fluid filled/mucus blocked sinus (arrow). (J-K) Hematoxylin and eosin-stained kidney biopsy from 1.II.1 showing mild tubular dilatations and a small glomerular cyst. Scale bars are 100 microns. (L-M) Growth curves of patients 1.II.1 and 1.II.2 showing progressive growth retardation with age. (N-P) Severe brachydactyly with shortened metacarpal bones. (Q) Pre-axial polydactyly of the right hand. (R) Pre- and postaxial polydactyly of the right foot and postaxial polydactyly of the left foot. (S) Chest radiograph showing pulmonary infiltrations (arrows). (T) Chest radiograph showing handlebar clavicles (arrow) and shortened horizontal ribs. (U) Pelvis radiograph showing a narrow pelvis with a coxa valga deformity. (V) Chest radiograph showing very short horizontal ribs and handlebar clavicles (arrow).

To identify genetic causes of ATD, SRPS and ATD-like ciliary chondrodysplasia phenotypes, we performed exome and targeted capture sequencing of known skeletal ciliopathy and PCD genes from affected individuals. In five individuals from four unrelated families, we found biallelic putative loss of function variants in *IFT74*. Sanger sequencing confirmed a biallelic loss of function variant in a clinically affected sibling in one of the families (Figs 1, 2 and S1 and Tables 1 and S1).

**Table 1:** Clinical Data.

Feature	Family 1		Family 2	Family 3	Family 4
Case	1.II.1	1.II.2	2.II.2	3.II.1	4.II.1
Ethnicity	Turkish		Turkish	Caucasian (British)	Caucasian
Current age	13 yrs (female)	9 yrs (male)	16 yrs (male)	Fetus (female)	Neonate (male), 37 weeks of gestation, deceased 7 days after birth
Clinical diagnosis	Jeune asphyxiating thoracic dystrophy / mucociliary clearance disorder	Jeune asphyxiating thoracic dystrophy / mucociliary clearance disorder	Jeune asphyxiating thoracic dystrophy / mucociliary clearance disorder	ATD / SRPS	SRPS/Jeune asphyxiating thoracic dystrophy
Genetic analysis performed	ES with CNV analysis followed by GS	Sanger sequencing	ES with CNV analysis	NGS panel	ES
*IFT74* variants (NM_025103.4/ ENST00000380062.9, GRCh38/hg38)	Homozygous exon 2 deletion g.26959922_26962977delinsTTATTATACTC	Homozygous exon 2 deletion g.26959922_26962977delinsTTATTATACTC	Homozygous exon 2 deletion g.26959922_26962977delinsTTATTATACTC	Heterozygous splice donor variant, c.974+4A>G (intron 12) + c.305+1664delG (intron 4) (g.26982280delG)	Homozygous splice donor variant c.789+2T>G (intron 10)
Short ribs/narrow chest	Yes	Yes	Yes	Yes	Restrictive pulmonary hypoplasia
Polydactyly	No	No	No	Yes, multiple polysyndactyly affecting all (both hands, both feet)	Affecting all 4 extremities
Disproportional short stature and skeletal features	-5.9 SD More pronounced with age, bowing of legs. Achondroplasia-like, short limbs, brachydactyly,	-4.9 SD Less severe than I.II.1; more pronounced with age, short limbs, brachydactyly	-8.5 SD (52 cm at birth, final height 118 cm) Short stature more pronounced with age, narrow thorax, short limbs, brachydactyly. Mild pectus excavatus, slanted acetabulum, hypermobility	Short proximal and distal limbs, severely short femurs/humeri. Short irregular growth plates, broad, poorly ossified cartilage-rich trabeculae, small scapulae and iliacs. 11 rib pairs, poorly ossified long, hand and foot bones and vertebral bodies.	Severely shortened extremities, thoracic narrowing.
Cranial and facial defects	Narrow foramen magnum	No	Flat nose, high arched palate.	Low set ears, broad nose, micrognathia. Midline cleft of upper lip. Posterior midline cleft palate, lobulated anterior tongue, tongue tie. Large head with wide skull sutures, absent brain falx and corpus collosum.	No
Heart defect	Mitral valve insufficiency, ASD II, PDA	No	Double aortic arch. (Older sib died of heart defect at 2.5 months)	Persistent left superior vena cava, AVSD, hypoplastic left heart, aortic atresia.	DORV, hypoplastic left ventricle
Renal disease	Progressive renal insufficiency	No	Hyperechoic kidneys, proteinuria chronic renal insufficiency, arterial hypertension	Cloacal abnormality with fusion of vaginal and bladder and bladder outflow obstruction. Dilated ureters and renal pelves; bilateral renal cystic dysplasia on histology.	No.
Liver Disease	No	No	No	Liver histology suggests probable ductal plate malformation.	No
Retinal disease	No	No	Retinitis pigmentosa	N/A (fetus)	Not tested
Obesity	No	No	Yes	N/A (fetus)	No
Intellectual delay	No	No	No	N/A (fetus)	N/A (lived only for 7 days as a preterm neonate).
Other features	No	No	Hypertension	Annular pancreas	No
Situs inversus	No	No	No	Intestinal malrotation.	No
Low nNO (normal >300nl/min; in PCD often > 77nl/min)	< 150 nl/min	< 150 nl/min	Not tested	N/A (fetus)	Not tested
Recurrent respiratory infections	Yes, more severe than 1.II.2	Yes	Recurrent bacterial infections and pneumonia in first 6 years	N/A (fetus)	N/A
Other respiratory features	Neonatal respiratory insufficiency in 1.II.1; Recurrent rhinosinusitis and persistent wet cough, more sever in 1.II.1 than I.II.2	Recurrent rhinosinusitis and persistent wet cough	Rhinosinusitis	N/A (fetus)	Severe respiratory insufficiency due to pulmonary hypoplasia
Otitis media	Yes, more severe in 1.II.1	Yes	Yes	N/A (fetus)	N/A (lived only for 7 days as a preterm neonate).
Motile cilia function	Reduced cilia numbers, very short cilia	Reduced cilia numbers, very short cilia, also after cell culturing	Reduced cilia numbers, very short cilia	N/A (fetus)	Not tested.

NGS, next-generation sequencing; ES, exome sequencing; GS, genome sequencing; SD, standard deviation of height; ASD, atrial septal defect; PDA, patent ductus arteriosus; AVSD, atrioventricular septal defect; DORV, double outlet right ventricle; nNO, nasal nitric oxide levels; N/A, not available.

In Family 1 and 2, routine filtering of the exome sequence [28–31] did not show any variants of interest. However, copy number variant analysis of next-generation sequencing data using ExomeDepth software [32] showed that the affected children carried a homozygous deletion of *IFT74* exon 2 (S1A and S1D Fig). This deletion is absent from the Database of Genomic Variants [33] and not present in ~10,000 exomes collected at Radboud University Medical Center (Radboudumc) Nijmegen. Genome sequencing performed in individual 1.II.1 confirmed a homozygous genomic re-arrangement with deep intronic breakpoints in intron 2 and intron 3, deleting exon 2 (Chr9:g.26959922_26962977delinsTTATTATACTC) (S1B and S1C Fig). Sanger sequencing confirmed the variant in a homozygous state in the clinically affected brother 1.II.2 while both parents of family 1 were found to be heterozygous carriers (Fig 1A). Uniprot reports three isoforms of *IFT74* that vary at their C-termini (Fig 1B and 1C). Exon 2 contains the predicted start codon for both isoforms and the patient deletion is predicted to delete the start codon and first 40 amino acids of all isoforms (Fig 1C). The first codon of exon 3 is a methionine giving potential for in-frame translational initiation at this point. Interestingly, a homozygous deletion of exon 2 of IFT74 with the same 5’ breakpoint but a slightly different deep intronic 3`breakpoint (Chr9:g.26959922_26962969delinsTTA) was previously identified in a neonate diagnosed with SRPS. RT-PCR confirmed absence of exon 2 from IFT74-cDNA while effects on protein level were not investigated [34]. In Family 3, a fetus with a SRPs phenotype was found to have compound heterozygous splice site variants, c.974+4A>G and g.26982280delG (c.305+1664delG) within *IFT74*. Both variants are absent from the exome variant server. c.974+4A>G lies within intron 12 and Alamut software predicts the creation of a novel splice site 4 bp after the original splice which would result in a frameshift allele. g.26982280delG affects a deep intronic position in the two major *IFT74* transcripts. Interestingly, it localizes to the -3 splice acceptor position (c.106-3delG) in a rare transcript ENST00000518614.5 that shares exons 1–4 with the major transcripts but has alternative exons 5 and 6 (Fig 1B). The protein coded by transcript ENST00000518614.5 shares a common 102 residue N-terminus with the two major isoforms followed by 44 unique residues before terminating in an alternative exon 6 (Fig 1C). As no RNA could be obtained from the fetus or the parents and the variants affect non-consensus splice site positions, we performed a minigene assay to test the effects of the variants on splicing. In the minigene assay, c.974+4A>G caused exclusion of exon 12 (41 bp) from the cDNA (S2A Fig). In IFT74 protein, this would be predicted to cause a 13 amino acid deletion followed by frameshift and a premature stop codon (c.Gln325?delfs*). Using sequence carrying the g.26982280delG variant in the assay resulted in inclusion of sequence corresponding to the alternative exon 5 of ENST00000518614.5, which was not observed when wild type sequence was used in the assay (S2B Fig). Our finding suggests that the g.26982280delG variant would prevent the formation of major isoforms in favor of the short ENST00000518614.5 isoform, which is not likely to support IFT-B particle formation and cilia assembly.

In Family 4, a newborn with SRPS or lethal ATD was found by exome sequencing to be homozygous for the substitution variant c.789+2T>G (Fig 1A). This variant affects the exon 10 consensus donor splice site and is predicted to abrogate the splice site, likely resulting in skipping of exon 10 which would result in an in-frame product with a 21 amino acid deletion after amino acid 263. Inclusion of the entire intron would result in an in-frame insertion of over 1500 amino acid, which seems unlikely. A shift of the splice site to a deeper intronic position could also result in an out of frame product. Unfortunately, no RNA was available from the family for verification.

### Phenotypic analysis of affected individuals

Family 1, a consanguineous family of Turkish descent living in Germany, displayed a spectrum of ciliopathy disease features. Individual 1.II.1 presented with a narrow thorax, brachydactyly and short extremities at birth (Fig 2A–2C, 2I–2L, 2N and 2S). Low thorax volume and recurrent respiratory infections prompted multiple hospital visits with an additional mucociliary clearance disorder clinically suspected. Disproportional short stature of increasing severity with age was noted with a current height at -5.9 SD (Fig 2L). Severe brachydactyly and development of severe bowing of the lower legs was noted (Fig 2C). The child developed mild proteinuria. Renal biopsy revealed mild tubular and glomerular dilatations with slightly increased interstitial fibrosis (Fig 2J and 2K). Renal disease progressed during late childhood. At age five, the patient presented with recurrent respiratory infections and nasal nitrous oxide below 200 ppm. Transmission electron microscopic (TEM) analysis of ciliated nasal polyp tissue showed reduced cilia number and cilia length with inconsistent loss of outer dynein arms and translocation of microtubule pairs. Diagnostic genetic analysis for chondrodysplasias, including achondroplasia and targeted next-generation sequencing gene panel analysis for all known ATD, Sensenbrenner Syndrome and PCD genes to date (Bioscentia, Germany) did not reveal any disease-causing variants. Her brother, individual 1.II.2, presented with a milder but similar phenotype of mildly narrowed thorax, short stature worsening with age, brachydactyly and recurrent respiratory infections but no renal disease has been detected (Fig 2C–2E, 2M and 2O).

Individual 2.II.2 was born as second child to a consanguineous Turkish family with a congenital heart defect (double aortic arch). The individual displayed a narrow thorax but had normal birth length (52 cm) and developed a progressive short stature and progressive limb shortening with brachydactyly (Fig 2F, 2G, 2O, 2P, 2T and 2U). An older sibling passed away in early infancy due to a congenital heart defect with no genetic testing performed. During early childhood, individual 2.II.2 suffered from chronic rhinosinusitis and recurrent chest infections which became less frequent from the age of 6 years on. He developed hyperechoic kidneys, proteinuria, and chronic renal disease as well as retinitis pigmentosa later in childhood.

Individual 3.II.1 was a fetus descending from non-consanguineous parents in the UK. Very short ribs, shorter long bones, polydactyly, cleft lip and palate, a complex congenital heart defect (persistent left superior vena cava, atrioventricular septal defect, hypoplastic left heart, aortic atresia), lobulated anterior tongue, absent brain falx and absent corpus callosum, cloacal malformation, cystic dysplastic kidneys (Fig 2H, 2Q, 2R and 2V) and pathognomonic radiological pelvis yielded a clinical diagnosis of ATD or SRPS.

Family 4 was a family living in North America with unknown consanguinity whose first-born child was prenatally diagnosed with ATD or SRPS due to polydactyly, short ribs, narrow thorax, and short long bones. He was born with a congenital heart defect (double outlet right ventricle). Postnatally, the diagnosis was confirmed by the classic ATD/SRPS pelvis appearance in x-ray. The child suffered from severe respiratory distress in the neonatal period requiring mechanical ventilation and subsequently passed away due to cardiorespiratory failure.

### Effects of the *IFT74* exon 2 deletion on ciliary ultrastructure

**Fig 3:**
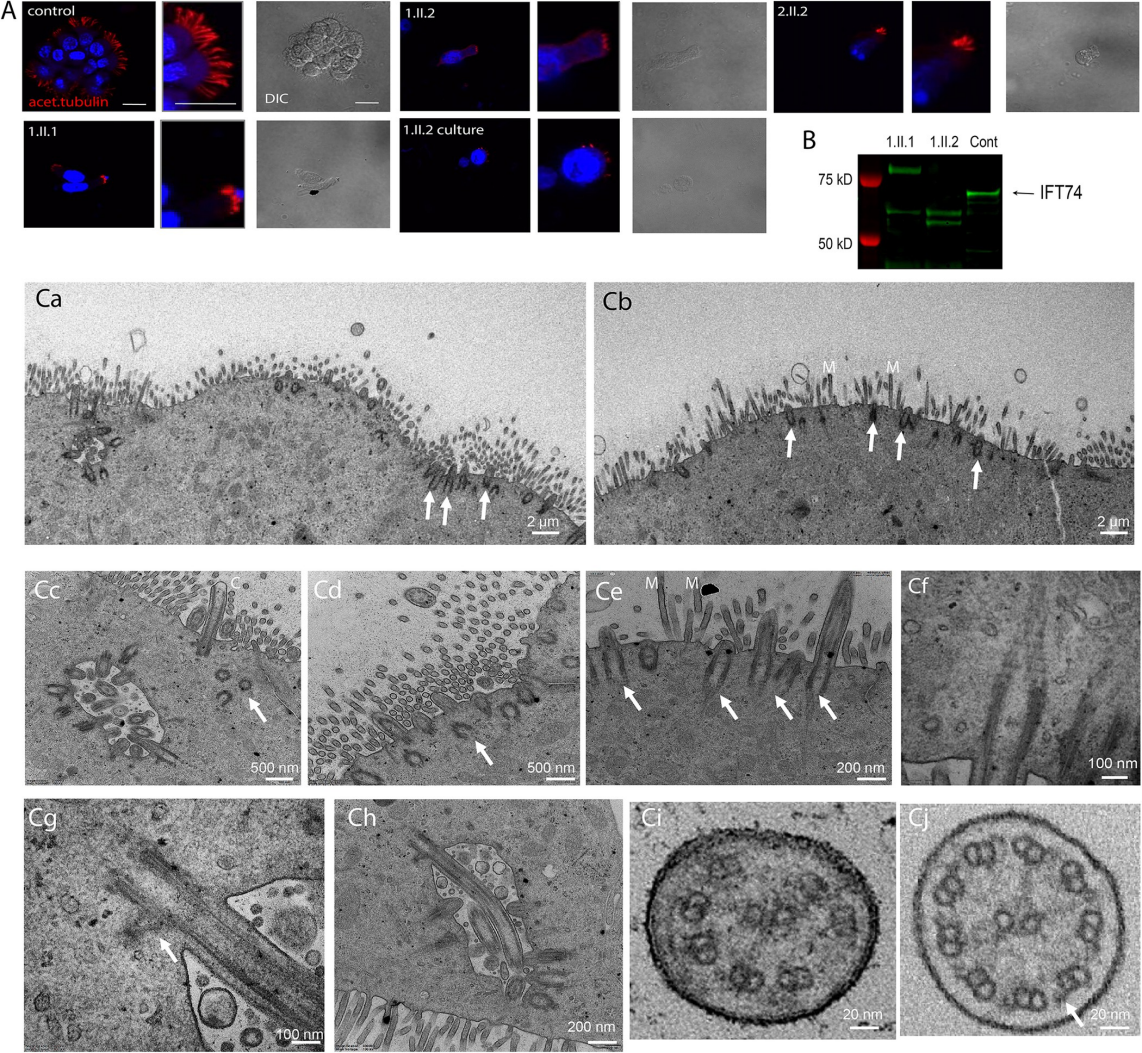
Patients with IFT74 Exon2 Deletion Have Motile Cilia Defects. (A) Immunofluorescence of respiratory epithelium from a healthy individual (control) and IFT74 exon 2 deletion affected individuals (1.II.1, 1.II.2, 2.II.2). 1.II.2 culture was a biopsy that was cultured for 6 weeks before fixation. Red (acetylated tubulin) marks ciliary axonemes. Blue (DAPI) marks nuclei. Scale bars in the first three images are 20 microns. Corresponding other images in panel are at the same scale. (B) Western blot of nasal biopsy samples. (C) TEM overview images of multiciliated nasal cells (**Ca**, individual 1.II.2 after cell culture; **Cb**, 1.II.1 native before cell culture) depicting very short cilia (arrows) mostly not even reaching the length of adjacent microvilli (M). **Cc**,**Cd**, close-up image of a ciliated nasal cell in individual 1.II.1 showing shortened cilia (**Cc**) and several undocked basal bodies below the cell surface (arrows). **Ce**, short cilia extending from normal docked basal bodes (arrows) in individual 1.II.1. **Cf**, normal appearing striated ciliary rootlet in 1.II.1. **Cg**, normal appearing striated basal foot in individual 1.II.1. **Ch**, example of a longer normally docked cilium in 1.II.1. **Ci**, ciliary cross sections example of individual 1.II.1 showing microtubule pair misarrangement with missing pairs and no apparent outer dynein arms, indicated with an arrow in a control cross section in **Cj**.

Family 1 clinical symptoms suggestive of PCD associated with *IFT74* exon 2 deletions prompted us to perform nasal brush biopsies (Fig 3 and S1–S4 Videos). Video microscopy of a biopsy from a control individual showed well ciliated respiratory epithelium with expected motility (S4 Video). Biopsy from 1.II.1 showed ciliated cells with altered motility (S1 Video). Biopsy from 1.II.2 showed sparse cilia with little motility (S2 Video), which did not improve after culture (S3 Video). Immunofluorescence analysis of the biopsies using acetylated tubulin as ciliary axonemal marker showed reduced numbers of very short motile cilia in all affected individuals as compared to controls. The shortened and sparse cilia phenotype persisted after six weeks of culture, excluding secondary effects due to infections **(**Fig 3A**).** TEM of the brushings from Family 1 confirmed the phenotype of very sparse cilia (Fig 3Ca and 3Cb). In contrast to the 10 micron length typical of normal cilia, *IFT74* mutant cilia were 0.5–1 microns long and failed to extend beyond the microvilli layer (Fig 3Cb). Basal body amplification and docking appeared unaffected in all three individuals with *IFT74* exon 2 deletion and no obvious defects affecting distal appendages or ciliary rootlets were visible (Fig 3Cc–3Ch). TEM cross sections showed that the *IFT74* exon 2 deletion yielded a variety of defects including loss of central pair or outer doublet microtubules, microtubule translocations and loss of microtubule integrity (Fig 3Ci) as compared to control cilia (Fig 3Cj).

To understand how the loss of exon 2 affects IFT74 structure, we performed western blot analysis of fresh nasal brushing samples from family 1. Brushings from control patients showed a band of ~69kD that likely represents full length IFT74. Affected individuals 1.II.1 and 1.II.2 lacked the 69kD band but had two or three smaller bands in the 50–60 kD range. Individual 1.II.1 also showed a larger band not seen in the control or her sibling 1.II.2 (Fig 3B). The source of the extra bands is not known.

### Generation of mouse Ift74 alleles

**Fig 4:**
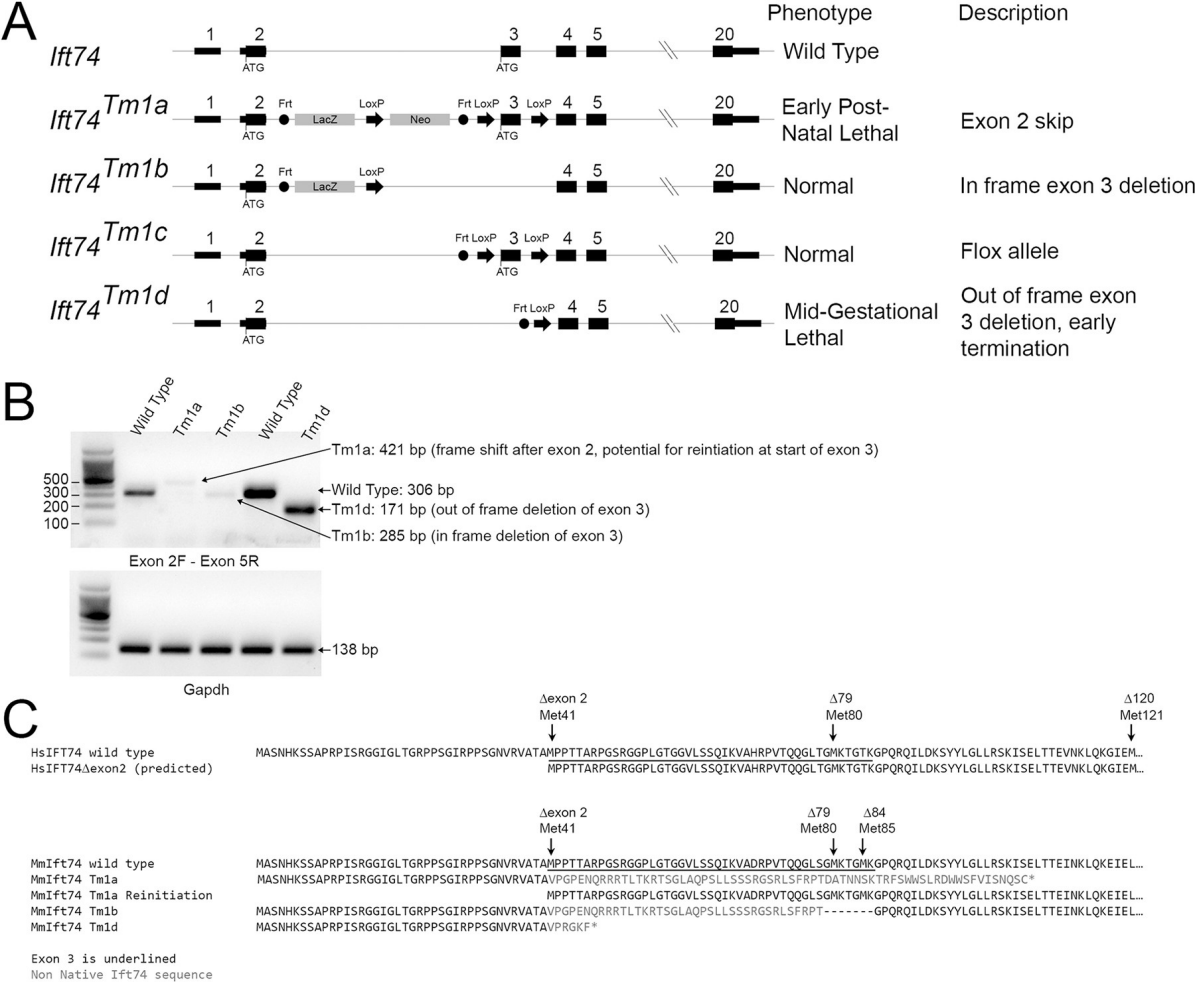
Mouse Alleles. (A) Diagram of the alleles used in this study. The normal start codon in exon 2 and the potential alternative start codon at the start of exon 3 are shown. FRT and LoxP recombination sites are represented by circles and arrows respectively. Exons and introns are not drawn to scale and the introns do not reflect changes that occur during recombination. (B) PCR amplification of cDNA generated from wild type, *Ift74*^*Tm1a*^, *Ift74*^*Tm1b*^ and *Ift74*^*Tm1d*^ variants using primers that span from exon 2 to exon 5. Note that alternative splicing produced small amounts of mRNA with the gene trap insertion removed from the *Ift74*^*Tm1a*^and *Ift74*^*Tm1b*^ alleles. The expected product was produced by the *Ift74*^*Tm1d*^ allele. Gapdh is included as a control for cDNA quality. Sequences of the products are included in S1 Data. (C) Predicted protein sequence of the alleles. Also see S1 and S2 Data files.

**Fig 5:**
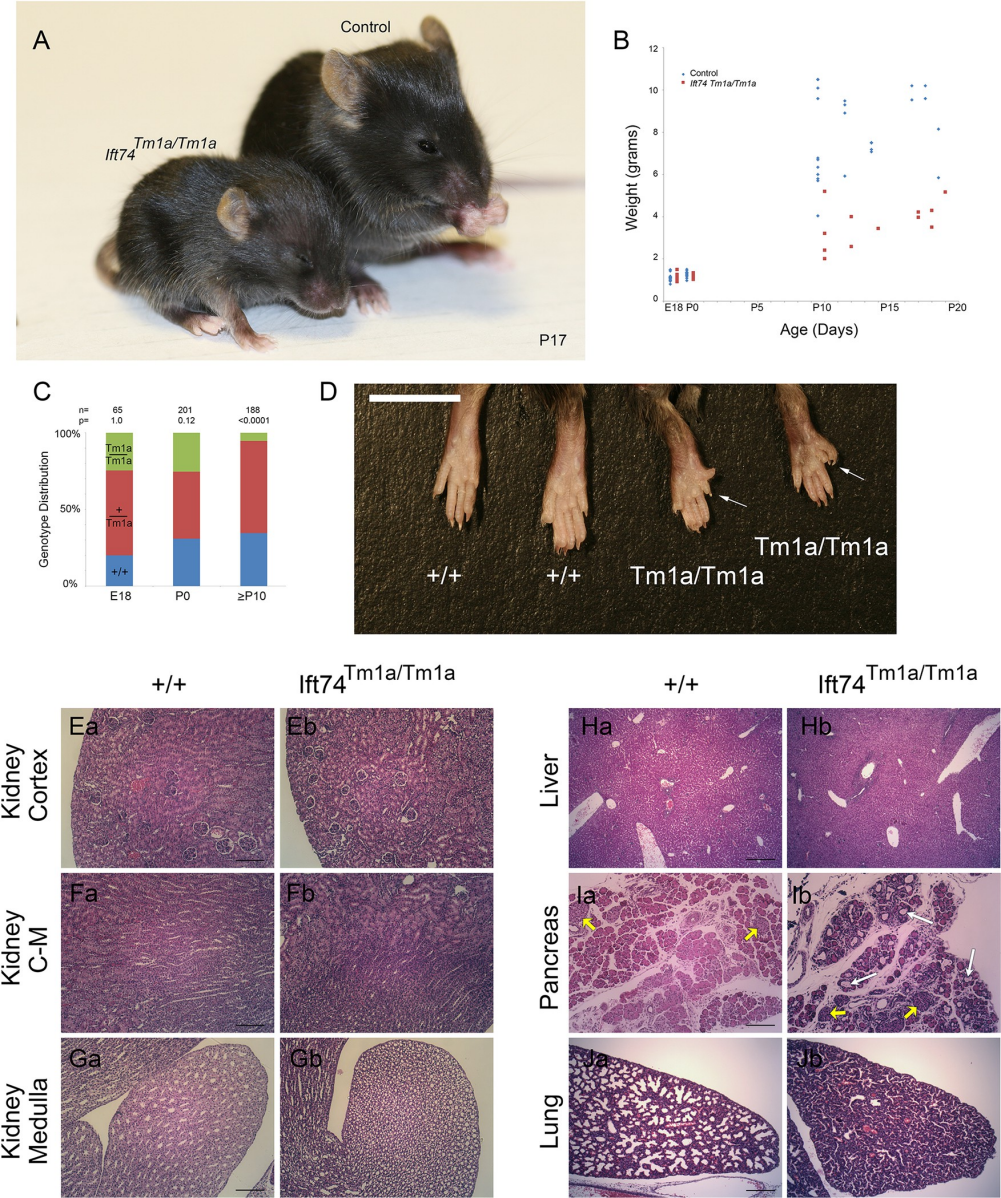
Ift74^Tm1a^ (Exon 2 Skip) Mutation Causes Post Natal Lethality. (A) Images of control and mutant mice at postnatal day 17. Note the smaller size and hydrocephaly in the mutant. (B) Weight of mice with respect to age. Each point represents a mouse at a particular age. Differences are significant with respect to genotype (P<0.0001, F test). (C) Genotype distribution at the day prior to birth (E18), the day of birth (P0) and at genotyping age (P10 to P21). Number of animals is listed along with the P value determined by Chi Square analysis is listed at the top. (D) Images of hindlimbs of controls and mutant animals. Note the extra digits on the mutants. (E-G) H&E Images of P15 kidney cortex (control Ea, mutant Eb), cortical-medullary boundary (control Fa, mutant Fb) and medulla at the tip of the papilla (control Ga, mutant Gb). Scale bars are 100 microns. (H) H&E Images of P8 liver (control Ha, mutant Hb). Scale bar is 200 microns. (I) H&E Images of P8 pancreas (control Ia, mutant Ib). Islets are marked with yellow arrows. White arrows mark examples of cysts. Scale bar is 100 microns. (J) H&E Images of P0 lung (control Ja, mutant Jb). Scale bar is 200 microns.

**Fig 6:**
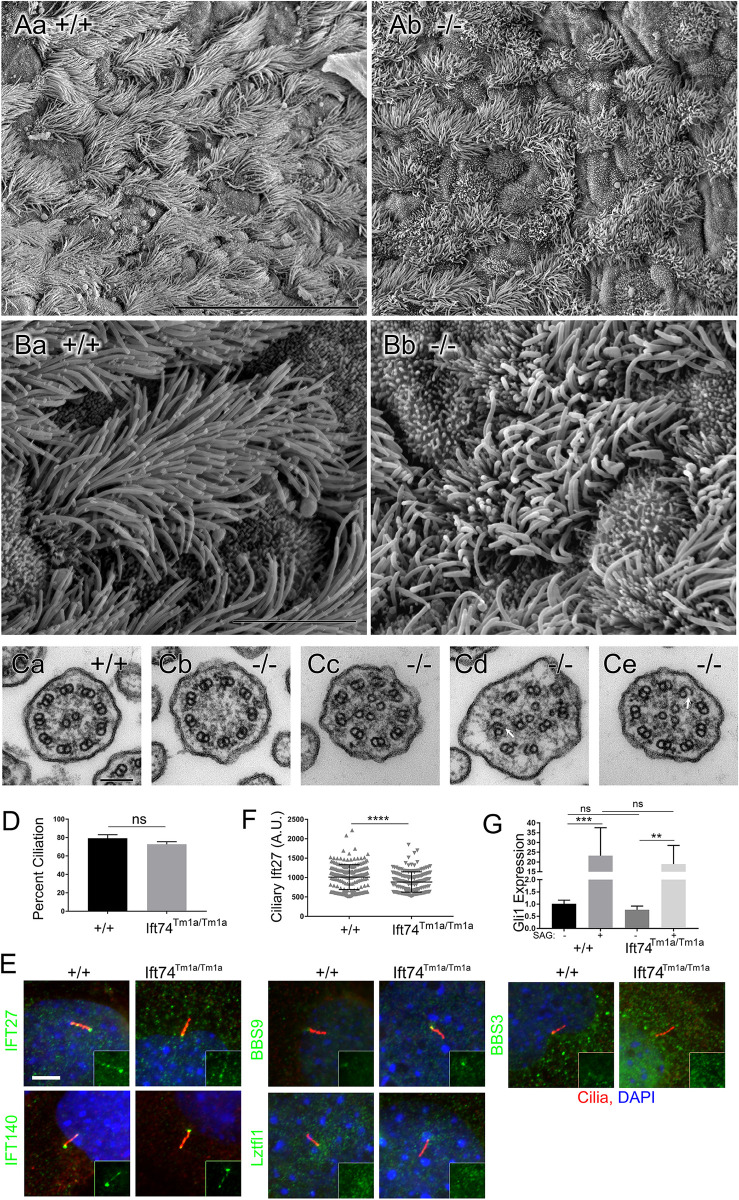
Ift74^Tm1a^ (Exon 2 Skip) Mutation Causes Motile Cilia Defects. (A, B) Scanning EM of control (Aa, Ba) and *Ift74*^*Tm1a*^ mutant (Ab, Bb) trachea. Note the uneven length of the cilia in the mutant. Scale bar in A is 30 microns; in B it is 5 microns. N = 4 mutant mice examined. (C) Transmission EM of cilia from control (Ca) and *Ift74*^*Tm1a*^ mutant (Cb-Ce) trachea. Scale bar in G is 100 nanometers. Note the absence of central pair in Cb, the extra microtubules in Cc-Ce and the open B tubules (arrow) in Cd and Ce. Quantification of ciliary defects is presented in S4 Data. (D) Percent ciliation in serum starved MEFs. N = 3 cell lines of each genotype, 100 cells counted per cell line. Difference is not significant (ns) by t-test. (E) MEFs labeled with the IFT antibodies (green) as listed on the left side. Cilia are labeled with Arl13b (red). Quantification of the pattern is presented in S5 Data. Insets show only the green IFT channel. (F) Quantification of Ift27 levels at the ciliary base (**** p<0.001 by t-test). (G) Induction of *Gli1* by SAG. Differences between controls and the *Ift74*^*Tm1a*^ mutants are not significant (ns). Induction of Gli1 by SAG was significant in both genotypes (**p≤0.01, *** p≤0.001 by two-way ANOVA).

**Fig 7:**
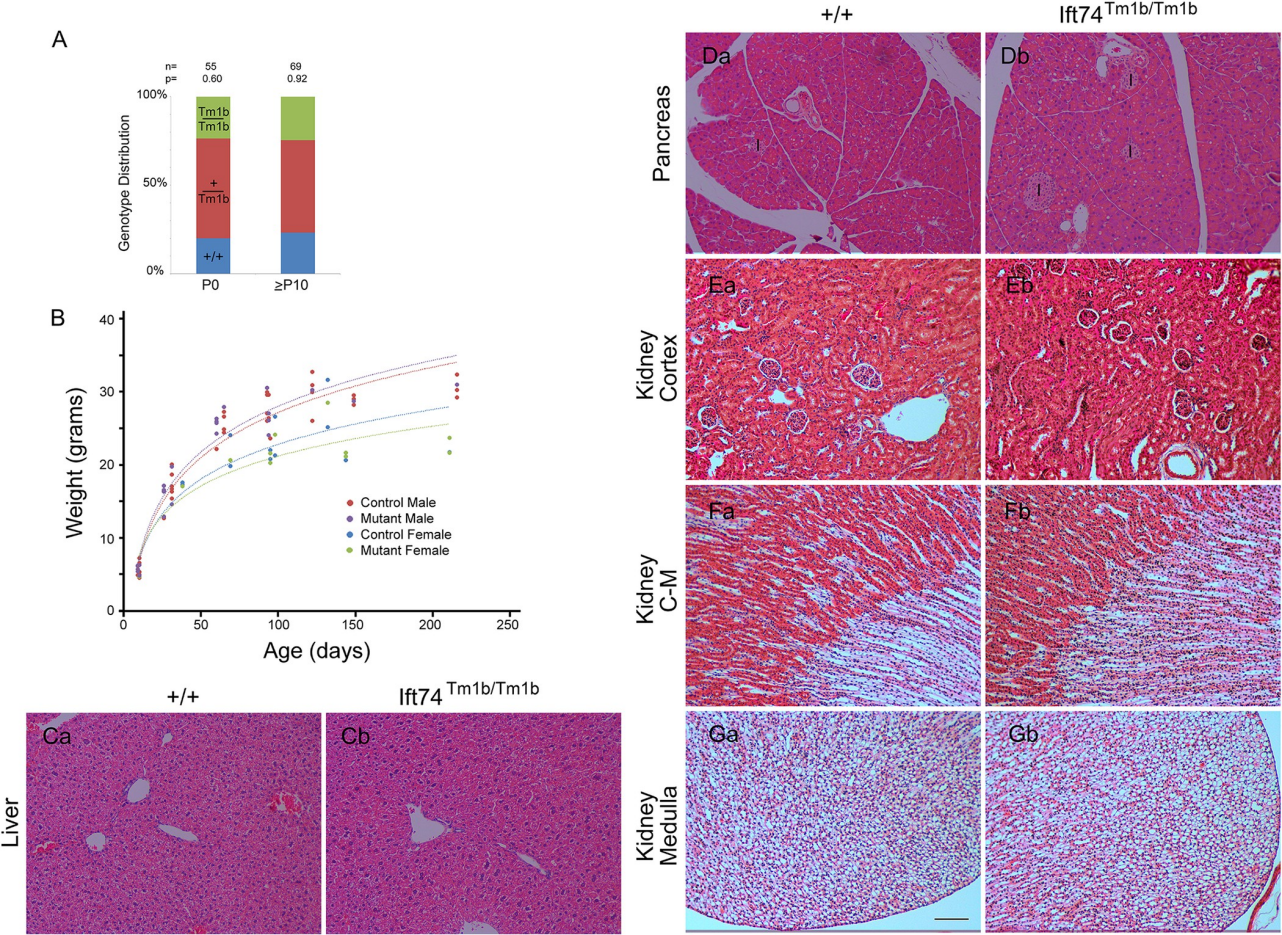
Ift74^Tm1b^ (In Frame Exon 3 Deletion) Mutation Results in No Detectable Phenotype. (A) Genotype distribution on the day of birth (P0) and at genotyping age (P10 to P21). Number of animals is listed along with the P value determined by Chi Square analysis is listed at the top. (B) Weight of mice with respect to age. Each point represents a mouse at a particular age. The same mouse may be represented by multiple points. Genotype did not influence weight within each sex (p>0.05, F test). (C) H&E Images of liver (control Ca, mutant Cb). (D) H&E Images of pancreas (control Da, mutant Db). Islets are marked with I. (E-G) H&E Images of kidney cortex (control Ea, mutant Eb), cortical-medullary (C-M) boundary (control Fa, mutant Fb) and medulla at the tip of the papilla (control Ga, mutant Gb). Scale bar is 100 microns and applies to images in C-G.

**Fig 8:**
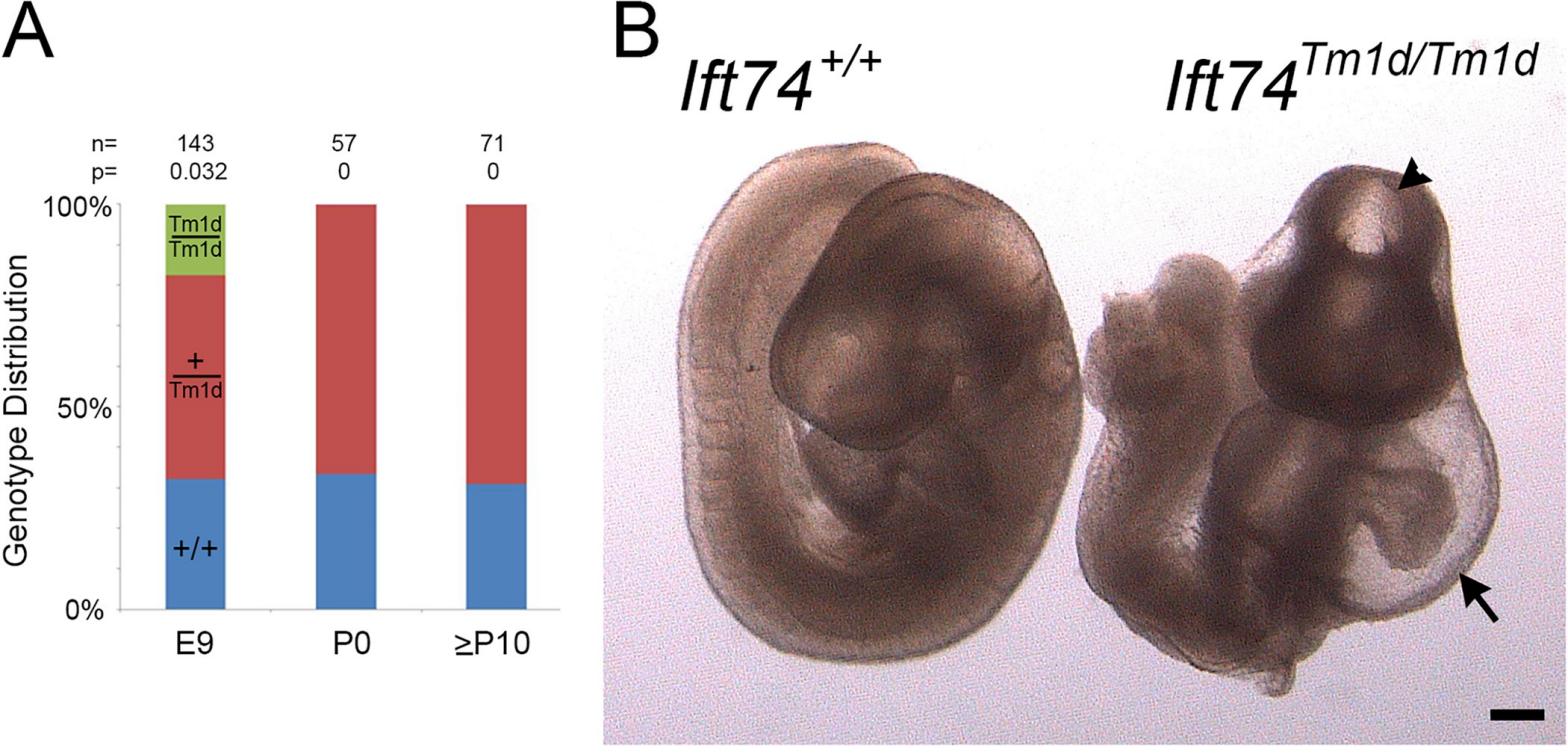
Ift74^Tm1d^ (Out of Frame Exon 3 Deletion) Mutation Causes Mid-gestational Lethality. (A) Genotype distribution at embryonic day 9 (E9), the day of birth (P0) and at genotyping age (P10 to P21). Number of animals analyzed along with the P value determined by Chi Square analysis is listed at the top. (B) Images of control and mutant mice at embryonic day 9. Note the malformed heart with pericardial effusion (arrow) and the abnormal head closure (arrowhead). Scale bar is 200 microns.

To characterize the function of Ift74 in mouse, we obtained the *Ift74*^*Tm1a*^ allele from Jackson lab (Figs 4, 5 and 6). This allele contains a promotor-less beta-galactosidase gene trap cassette and a neomycin selectable marker inserted into intron two along with Cre recombination (LoxP) sites flanking exon three (Fig 4A). The beta-galactosidase-neomycin insert in intron 2 is flanked by Frt recombination sites, which were removed with FlpE recombinase to create a floxed allele (*Ift74*^*Tm1c*^). Exon 3 is flanked by LoxP sites, which can be excised with Cre recombinase. Recombining with Cre before the FlpE recombination, removed exon 3, but left the beta-galactosidase gene trap cassette creating *Ift74*^*Tm1b*^ (Figs 4 and 7). Recombining with Cre after FlpE recombination removed exon 3 leaving only Frt and a Cre recombinase sites in the intron between exons 2 and 4 creating *Ift74*^*Tm1d*^ (Figs 4 and 8). The Tm1a allele is expected to be a null or strong hypomorphic allele due to the gene trap cassette terminating transcription after exon 1. The Tm1b allele retains the gene trap cassette and lacks exon 3 suggesting that it should be at least as strong as the Tm1a allele. The Tm1c (flox) allele is expected to be normal and the Tm1d allele is designed to produce an out of frame splice of exon 2 to exon 4 and should be a null allele. However as will be described in the following sections, the Tm1a and Tm1b phenotypes were not as expected (Table 2).

**Table 2:** Summary of Mouse Alleles.

*Ift74* allele	Genomic Locus	Predicted Protein	Mouse Phenotype	Cilia Phenotype
Tm1a	All exons intact, has gene trap insertion	Missing exon 2-coded residues, protein likely initiates at start of exon 3 (Met 41)	Post natal lethal with polydactyly, hydrocephaly, and growth restriction	Primary cilia on cultured fibroblasts are mostly normal, Motile cilia in the mouse are defective
Tm1b	Exon 3 is deleted, has gene trap insertion	In frame deletion of exon 3	Viable, with no detectable phenotype	Primary cilia on cultured fibroblasts are normal
Tm1c	Exon 3 is floxed	No expected effect	Viable, with no detectable phenotype	Not examined
Tm1d	Exon 3 is deleted	Out of frame deletion of exon 3, early termination after exon 2	Mid-gestational lethal, with malformed heart	Primary cilia do not form on cultured fibroblasts

### *Ift74*^*Tm1a*^ causes postnatal lethality with polydactyly and hydrocephaly

Crosses between *Ift74*^*Tm1a*^ heterozygotes produced expected ratios of homozygous mutant animals on the day prior to birth (E18) and on the day of birth (P0) (Fig 5C). At E18 all mutants were alive and healthy appearing, with polydactyly being the only obvious phenotype (Fig 5D). On P0, most of the mutants were dead by collection time. However, some survived and could be identified by the presence of polydactyly. Mutants usually lacked a milk spot, indicating a failure to nurse. In typical large litters, none of the mutants survived past day 0 but with selective culling to reduce litter size to four, some mutants could survive past P0 with the oldest surviving to P20. The survivors were typically about half as big as littermates and developed hydrocephaly, necessitating euthanasia (Fig 5A and 5B).

Sixteen E18 and 23 P0 mutants were analyzed by microCT and necropsy (S2 and S3 Tables [spreadsheets]). The prevalent phenotype observed by microCT was hindlimb polydactyly with most animals showing at least unilateral polydactyly. In addition, examples of duplex kidney and hydronephrosis were observed. Cardiac malformations were rare with only one example of a right aortic arch and a possible ventricular septal defect observed. Necropsy of P0 animals showed similar findings with a high penetrance of hindlimb polydactyly along with four examples of forelimb polydactyly. Duplex kidney was observed in three animals along with one example of left lung isomerism and one example of malaligned sternal vertebrate. Significant disturbances of the left-right axis were not observed by either microCT or necropsy.

Histological analysis did not detect any evidence of cyst formation in the kidney or liver (Fig 5E–5H). In contrast, every post P0 animal examined had an abnormally small pancreas. Histological analysis showed cyst formation within the exocrine ducts with fibrotic material surrounding the cystic ducts (Fig 5I). Lungs collected from two of four P0 animals showed reduced saccule area and abnormally thick walls suggesting hypoplasia (Fig 5J). The lung hypoplasia may be responsible for the inability of some animals to survive postnatally.

Hydrocephaly suggests motile cilia dysfunction, so we examined tracheal cilia by scanning and transmission EM (Fig 6). When examined by scanning EM, wild type tracheas showed uniform ciliation. In contrast, the cilia in all mutants were shorter and unequal in length as compared to littermate controls. Mutant cilia also appeared thicker and less uniform in diameter than controls and had occasional bulges at the tip (Fig 6A and 6B). Transmission EM showed a variety of low frequency defects in cross sections of mutant cilia. These defects included absent central pair microtubules, extra central pair microtubules, displaced doublets, and failure of the B-tubule to fully connect to the A tubule (Fig 6C and S4 Data).

MEFs derived from the *Ift74*^*Tm1a*^ mutant animals were able to ciliate as well as controls (Fig 6D) and staining with IFT markers, Ift140, Bbs9, Lztfl1 and Bbs3 did not show any differences from controls (Fig 6E and S5 Data). The only detectable phenotype was a slight reduction in the amount of Ift27 localized to the base of the cilium (Fig 6E and 6F and S5 Data). Ift27 is thought to connect to the IFT particle through Ift74 and Ift81 and so this may reflect the reduction of Ift74 in the cells [35]. This reduction does not appear to be highly significant as induction of Gli1 expression by SAG treatment was similar in mutants and controls (Fig 6G). Likewise, we did not observe any significant differences in localization for the centriolar proteins CBY and CP110 in mutant MEFs compared to control MEFs (S4 Fig).

### *Ift74*^*Tm1b*^ does not cause detectable phenotypes

Crosses between *Ift74*^*Tm1b*^ heterozygotes produced expected ratios of homozygous mutant animals on the day of birth (P0) and when genotyped at P10 or later (Fig 7A). Homozygous mutants were similar in weight to controls (Fig 7B), had no observable phenotypes and bred normally. Histological analysis detected no cysts or other pathology in the kidney, liver, or pancreas (Fig 7C–7G). MEFs ciliated normally. These findings are similar to what was reported by the KOMP/EUCOMM phenotyping project (http://www.mousephenotype.org/data/experiments?geneAccession=MGI:1914944) where they reported abnormal blood glucose levels and behavioral differences but no structural birth defects or alterations in viability in this allele.

### *Ift74*^*Tm1d*^ caused mid-gestational lethality

**Fig 9:**
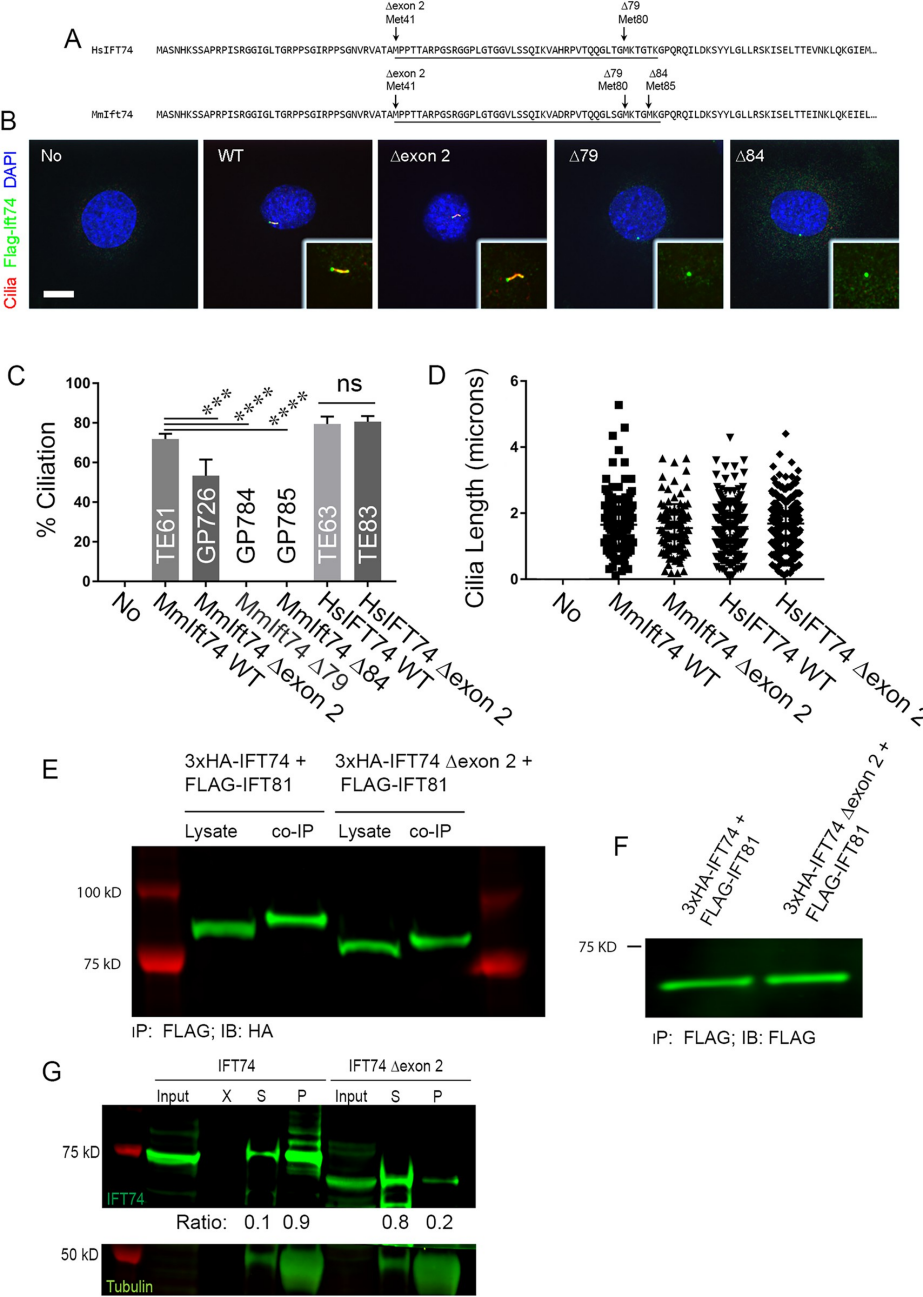
Ift74^Tm1d^ MEFs Rescued with N-terminal Deletion Alleles. **(A)** Alignment of human IFT74 and mouse Ift74 with the positions of the methionines marked. These methionines were the initial codons in the expression vectors used in the latter panels. Exon 3 is underlined. **(B)** MEFs stained for cilia (Arl13b, red), Ift74 (Flag, green) and DAPI (blue). 20615.4T (*Ift74*^*Tm1d/Tm1d*^) MEFs derived from an E9 mutant embryo was completely unable to ciliate (No). Ciliation was rescued with wild type mouse *Ift74* (TE61) and *Ift74* lacking exon 2 (GP726, Δexon2). Mouse *Ift74* initiating translation at Met 3 (GP784, Δ79) and Met 4 (GP785, Δ84) was unable to rescue ciliation. Insets are 2X enlargements of the cilia/centrosome regions. Images are maximum projections of 3 image Z stacks taken at 0.2-micron intervals. Scale bar is 10 microns. **(C)** Quantification of percent ciliation of the cells shown in panel B and in cells transfected with wild type human *IFT74* (TE63) or human *IFT74* lacking exon 2 (TE83, Δexon2). Mouse *Ift74* lacking exon 2 was less effective at rescuing ciliation (***p = 0.004) but no difference was seen with human *Ift74* lacking exon 2. Mouse Ift74 lacking sequences upstream of the third and fourth methionines did not rescue (****p<0.0001). N = 100 or more cells counted from 3 independent experiments. Data was compared using one-way ANOVA. **(D)** Cilia length in the same cells as described in panel C. No differences were seen in cilia length between any of the rescued lines that ciliated using one-way ANOVA. N = 100 or more cilia measured per line. **(E)** Co-Immunoprecipitation of wildtype 3xHA-IFT74 and 3xHA-IFT74 lacking exon 2 with Flag-IFT81, showing similar amounts of IFT74 and IFT74Δexon2 precipitated with IFT81. Co-IP was performed using anti Flag antibody and the blot stained with anti-HA antibody. **(F)** IFT81 expression control for the co-immunoprecipitation shown in (E) showing similar expression levels in cells expression wildtype or mutant IFT74. **(G)** IFT74-tubulin pulldown revealing decreased tubulin binding of IFT74 lacking exon 2 compared to wildtype IFT74. Tubulin was pelleted by ultracentrifugation and pellet and supernatant samples probed for presence of tubulin and IFT74. The majority of tubulin as well as wildtype IFT74 was found in the pellet after centrifugation, indicating IFT74-tubulin binding while IFT74 lacking exon 2 was detected predominantly in the supernatant indicating a failure to bind tubulin. The ratios of IFT74 in the pellet and supernatant are listed below the IFT74 western blot. Also see S3C Fig.

Crosses between *Ift74*^*Tm1d*^ heterozygotes produced no homozygous mutant animals that survived to birth (Fig 8A). Other complex B null variants cause lethality starting at ~E9 [36]. At E9, homozygous *Ift74*^*Tm1d*^ mutant embryos were slightly underrepresented (expected 36, obtained 26, P = .032). The mutants were all smaller than littermates, most failed to complete embryonic turning, had head closure defects and showed abnormal heart development with large pericardial effusions (Fig 8B). Most showed no evidence of heartbeat, and we were able to generate fibroblast cell lines from only one of seven embryos attempted while control littermates produced cell lines in all cases. The lack of heartbeat and failure to generate cell lines suggests that most embryos were already dead at the time of collection. Fibroblasts derived from the one mutant animal were completely unable to ciliate, but this could be rescued by transfection with Flag-tagged Ift74 (Fig 9A–9D).

### Complex splicing alters the *Ift74* alleles

As described in previous sections, the phenotypes of the Tm1a and Tm1b alleles did not match expectations. The Tm1a retains all coding information, but it contains a genetrap insertion that would be expected to terminate transcription early in the gene and produce a null allele. Ift74 is an integral part of IFT-B and loss of function would have been expected to cause a mid-gestational lethal phenotype like other critical IFT-B genes [36]. However, the observed phenotype was significantly milder with death in the post-natal period. The Tm1b allele lacks exon 3 and carries a gene trap insertion that should strongly reduce transcription beyond exon 2. Again, the loss of exon 3 and the gene trap would be predicted to produce a null phenotype, but no abnormalities were observed, and the mice were adult viable. To understand the causes of these unexpected phenotypes, we isolated mRNA from fibroblasts derived from each of the lines and analyzed the message structure around the insertion site by reverse transcriptase PCR (Fig 4B). Amplification of wild type cDNA with an exon 2 forward primer combined with an exon 5 reverse primer produced a 306 bp product with the expected sequence (S1 Data). Using the same primers on cDNA derived from the Tm1a allele produced a small amount of a 421 bp product. The sequence of this product indicated that it contained exon 2 and 115 base pairs of the gene trap vector fused to exon three (S1 Data). Translation of this message would go out of frame in the gene trap sequence (Fig 4C). However, the first codon of exon 3 specifies methionine. This methionine codon is in the original reading frame potentially allowing for reinitiation of translation. Thus, it is likely that the Tm1a allele produces a protein initiated at the first methionine of exon 3 and missing the residues coded by exon 2. This will be referred to as an exon 2 skip. Amplification of cDNA from the Tm1b allele produced a small amount of a 285 bp product which lacked exon 3 but retained part of the gene trap cassette (S1 Data). The predicted translated protein has the 45 exon 3 residues from Met41 to Lys86 replaced with 38 residues derived from the gene trap cassette. This will be referred to as an in frame exon 3 deletion. The Tm1d allele produced the expected 171 bp product where exon 2 was fused out frame to exon 4. This will be referred to as an out of frame exon 3 deletion. Predicted sequence of the resulting proteins is in Fig 4C and S2 Data, the sequence of the PCR products is in S1 Data, and a summary of the phenotypes and alleles is in Table 2.

### Exon 2 deletion impairs tubulin binding

To examine the importance of the Ift74 N-terminus to primary cilia formation, we transfected *Ift74* null (*Ift74*^*Tm1d/Tm1d*^) MEFs with expression constructs where translation would start at the normal methionine or at one of the three methionines in exon 3 (Fig 9A–9D). The parental null MEFs are unable to ciliate. Rescue with Flag-tagged wild type mouse *Ift74* restored normal length cilia to ~70% of cells (Fig 9B–9D). Rescue starting at the second methionine (Δexon2) was also fairly effective (~50%) but rescue starting at the third (Δ79) or fourth (Δ84) methionine was ineffective with no ciliated cells being found. The latter two constructs were expressed and localized to a spot near the nucleus, which is likely the mother centriole (Fig 9B). Transfection with human *IFT74* and *IFT74*^*Δexon2*^ was able to restore the ability to form normal length cilia to the null cells (Fig 9A, 9C and 9D). This observation indicates that IFT74 translated from the methionine at the beginning of exon 3 is at least partially functional.

To determine if the deletion of exon 2 affected the ability of IFT74 to be incorporated into the IFT particle, we co expressed HA-tagged CBY (centriolar protein used as a negative control) or IFT-B subunits, IFT81, IFT46 and IFT52, with Flag-tagged IFT74 or IFT74^Δexon2^ and immunoprecipitated with HA antibody. As expected, CBY did not precipitate any IFT74 or IFT74^Δexon2^ while IFT81, IFT46 and IFT52 similarly precipitated both IFT74 and IFT74^Δexon2^ (Figs 9E, 9F, S3A and S3B) indicating that the deletion of exon 2 does not greatly affect the ability of IFT74 to interact with other IFT-B proteins.

Structural studies indicate that the coiled-coil domain of IFT74 interacts extensively with IFT81 and together the IFT74/IFT81 N-termini form a tubulin binding site for transport of this major axonemal protein into cilia. The tubulin-binding site of IFT74 is thought to be coded by the N-terminal basic region [20], which is partly missing from IFT74^Δexon2^. Since IFT81 is thought to be the major tubulin binding site with IFT74 providing stability to the binding [20], we used an *in vitro* assay without IFT81 being present (Fig 9G). To do this, we incubated *in vitro* translated full length IFT74 or IFT74^Δexon2^ with purified bovine tubulin. After centrifugation, the relative amounts in the supernatant and pellet were compared. In the absence of microtubules, both IFT74 and IFT74^Δexon2^ were found in the supernatant indicating that they are not aggregated. As expected, microtubules pelleted. When microtubules were added to translated IFT74 or translated IFT74^Δexon2^ and the solution centrifuged at 100,000 g, most of the full length IFT74 sedimented with the microtubules whereas IFT74^Δexon2^ was mostly found in the supernatant. A similar assay with lower g forces confirmed that IFT74^Δexon2^ did not sediment as well as full length and showed that IFT74^Δ79^ and IFT74^Δ120^ did not sediment with microtubules (S3C Fig).

## Discussion

IFT74 is a subunit of the IFT-B complex [37] and in *Chlamydomonas* it is required for ciliary assembly [21,38]. In mammals, it was initially called CMG1/IFT71 and shown to localize to cilia on endothelial cells [39]. Human and mouse IFT74 genes are predicted to encode two main isoforms of 600 and 372 residues in humans and 600 and 388 residues in mouse. The isoforms share common N-terminal ends and in humans are identical for the first 351 amino acids and in mouse for 352 amino acids. The N-terminal ~90 residues is rich in lysines and arginines and is thought to work with IFT81 to form a tubulin binding domain facilitating most tubulin transport during ciliary assembly [20,21]. The basic region is followed by a helical SMC domain, which facilitates interactions with multiple other IFT-B subunits. Most extensive interactions are seen with IFT81, which are distributed along the non-basic region of the protein, but structural analysis shows interactions with IFT70, IFT88, IFT46, IFT22, IFT25 and IFT27 indicating that IFT74 is central to IFT particle structure [40,41].

In this work, we identified four human *IFT74* disease-causing variants and characterized three mouse *Ift74* alleles. The mouse phenotypes ranged from a severe mid gestational lethal phenotype in the *Ift74*^*Tm1d*^ out of frame exon 3 deletion allele, a post-natal lethal phenotype in the *Ift74*^*Tm1a*^ exon 2 skip allele, to no detectable phenotype in *Ift74*^*Tm1b*^ in frame exon 3 deletion allele. In the *Ift74*^*Tm1d*^ allele, the mid gestational lethal phenotype occurred at embryonic day nine when the mice presented with severe cardiac malformations. This phenotype is similar to other null alleles of critical *IFT-B* genes like *IFT88* [36]. The severe cardiac phenotype is likely to reflect the importance of cilia to heart development where it plays critical roles in setting up the left-right axis and in regulating Hedgehog signaling for development of the chambers and vasculature [13]. In humans, equivalent heart development would occur in the first trimester, making it unlikely that strong alleles of critical ciliary assembly genes will be identified in viable or later-term lethal patients. The *Ift74*^*Tm1b*^ allele, which does not produce a phenotype, was predicted to be null. However due to unexpected splicing, it results in an in-frame replacement of the third exon with a similar number of codons derived from the targeting vector. The residues that are missing are from an unstructured domain that is thought to be part of the tubulin binding domain [20] but are clearly not critical to protein function. Conversely, the *Ift74*^*Tm1a*^ exon 2 skip allele must affect protein function as these animals have significant phenotypes including growth restriction, hydrocephaly, polydactyly, and post-natal lethality. In mRNA isolated from these mice, the first coding exon is missing, which is likely compensated by translation initiation at a later methionine codon. Interestingly, the first triplet of the second coding exon specifies methionine. *In vitro* studies suggest that protein initiated at this codon is functional for primary ciliary assembly in fibroblasts (Fig 9). This is consistent with the mouse phenotype where motile cilia are affected but only subtle primary cilia phenotypes are observed.

Similar to what we observed in mouse, the human variants that we and others recovered in *IFT74*, result in a range of phenotypes. Most strikingly, we identified three individuals from two families (Families 1 and 2) in whom the first coding exon was deleted. A comparable deletion with identical 5’ but slightly different 3`intronic breakpoints was independently identified in a single patient [34]. The breakpoints identified in these exon 2 deletions do not affect the antisense *IFT74* gene or other genes within the genome. Ensembl indicates a CFTC site in the deleted region. Effects of CTCF site deletions are difficult to assess. However, it seems unlikely that deletion of this site contributes to the motile cilia phenotype observed in the human patients as the motile cilia phenotype is reproduced in the mouse model lacking the CTCF genomic deletion. We predict this deletion will produce an N-terminally truncated protein identical to what is produced by the mouse *Ift74*^*Tm1a*^ exon 2 skip allele. Post-natal growth restriction was observed in the mouse and the patients presented with short stature that became more pronounced with age. This phenotype not typically observed in ATD [25] where body length is often short at birth but catch-up growth is normal except for cases with end-stage kidney disease. In addition to the short stature, the patients presented with recurrent respiratory infections. Light and electron microscopy revealed significant defects in the motile cilia of the respiratory tract. These defects persisted after respiratory cell culture suggesting that it is a primary defect and not secondary to infection. In line with motile cilia defects resulting from IFT74 exon 2 deletion, the neonate described in [34] presented with extensive airway secretion problems but motile cilia analyses were not performed (personal communication with G. Grigelioninene and A Hammersöj, Karolinska University Hospital, Stockholm, Sweden). The *Ift74*^*Tm1a*^ mice developed hydrocephaly, which is a common phenotype of motile cilia defects in mice. Furthermore, scanning EM of the trachea of the affected mice showed shorter and fewer cilia and TEM showed a variety of axonemal defects.

Individual 1.II.1 showed proteinuria and kidney biopsy revealed mild tubular and glomerular dilations in early childhood but progressed to end-stage kidney disease. Individual 2.II.2 likewise suffers from reduced renal function and cystic dysplastic kidneys were observed in fetus 3.II.1. Cystic kidney disease, with associated liver and pancreas cysts and fibrosis, are common phenotypes in mice with cilia dysfunction. However, we did not observe kidney or liver phenotypes in the mouse although the pancreas was small and mildly cystic and fibrotic. *Ift74*^*Tm1a*^ mice typically presented with polydactyly. This is typically observed in about 10% of ATD patients but was not observed in any of the patients with an exon 2 deletion we studied. Retinal degeneration was observed in patient 2.II.2. This is common in mice with IFT-B variants [42] but we did not examine the retinas of the *Ift74*^*Tm1a*^ mice.

The individual in Family 3 was compound heterozygous for a splice donor variant c.974+4A>G and the intronic variant g.26982280delG (c.305+1664delG). Minigene splicing analysis suggests that the c.974+4A>G variant causes exon 12 to be skipped leading to a 13 amino acid deletion followed by frameshift and a premature stop codon. Variant g.26982280delG is unusual in that it is located deep in the intron of the major transcripts and would not be expected to be pathogenic. However, it is located at c.106-3delG in a rare transcript that uses alternative exons 5 and 6. Minigene analysis indicates that this variant shifts the balance to make more of the rare transcript and less of the major transcripts. The rare transcript produces a very short protein that is not likely to be functional for IFT-B particle assembly.

In Family 4, a newborn (4.II.1) with SRPS or lethal skeletal ATD phenotype, carried the variant c.789+2T>G. This variant, which is predicted to alter the exon 10 donor splice site, is likely to cause a 21 amino acid deletion after amino acid 263. Alternatively, it could result in the inclusion of the intron leading to an insertion of >1500 amino acids into the open reading frame, which seems unlikely. Regardless, the deletion or insertion would occur at a site where IFT74 has crosslinks to IFT81, IFT70 and the IFT25/IFT27 dimer making it likely that the variant would affect the ability of the protein to be incorporated into the IFT particle. The severe lethal ATD/SRPS phenotype caused by this variant suggests that it is a strong hypomorph.

Eleven human cases carrying biallelic *IFT74* variants have been published to date: one ATD / SRPS case, three BBS cases, five JBTS cases, and two cases with asthenozoospermia (isolated male infertility) (S1 Table). The ATD / SRPS case interestingly carried an exon 2 deletion comparable to our families 1 and 2 and while no motile cilia function testing was performed, personal communication with the authors revealed the patient presented with additional PCD-like respiratory problems in addition to thoracic dysplasia [34]. The three BBS cases carry the same splice site allele, c.1685–1G>T, which is expected to affect splicing of intron 19. In one case, the variant was homozygous [43] while in the other two cases, the splice site variant was in trans to a heterozygous deletion of exons 14–19 [44] or a frameshift variant, c.371_372del [45]. Disrupting splicing of intron 19 could potentially result in the production of protein consisting of 561 IFT74 residues followed by an extension of some number of non-IFT74 residues, although more disruptive outcomes such as message or protein instability are possible. The deletion of exons 14–19 would be expected to produce a protein of 351 normal residues with an unknown C-terminal end while c.371_372del is expected to cause a frameshift at Gln124.

The same Gln179Glu substitution variant (c.535C>G) was identified in five JBTS cases. One family, with two affected individuals, also carries a c.92delT variant that is expected to terminate translation at codon 285. The second family carries a c.306-24A>G variant that is expected to delete Ser103 to Arg135. The third family had c.85C>T variant in trans that would be expected to cause termination after codon 29 [46] while in a in a fourth family, Gln179Glu was identified together with c.853G>T, which would cause termination at codon 285 [47].

Additionally, two siblings with isolated male infertility were found to be homozygous for c.256G > A, which mutates Gly86 to Ser and changes the last nucleotide of exon 3. Changing the last nucleotide of exon 3 could potentially affect splicing. RT-PCR analysis showed that the variant did indeed cause imprecise splicing as a smear of amplification products was observed above and below the expected product. The two most abundant products were a normally spliced message with the Gly86Ser variant and one containing a 10-codon in-frame deletion between Leu77 and Gly86, but other splice variants were found [48]. The Gly86Ser variant is within the unstructured basic domain and is not likely to have a serious effect on the protein. The 10 amino acid deletion could be pathogenic, but it is within a region deleted in our phenotype-less *Ift74*^*Tm1b*^ mouse, indicating that this is not critical sequence. The failure to splice properly could reduce the amount of functional protein below a level critical for sperm development, but not low enough to affect other cilia. Observing male infertility in human patients is consistent with results in mouse where targeted deletion of *Ift74* in the testes results in sperm tail assembly defects and male infertility [49].

Human patients with variants in IFT-B encoding genes have been previously described for the subunits IFT27 (BBS) [50,51], IFT52 (SRPS) [52,53], IFT54/TRAF3IP1 (Senior-Loken syndrome) [54], IFT56/TTC26 (biliary, renal, neurologic, and skeletal syndrome) [55], IFT57 (orofaciodigital syndrome) [56], IFT80 (SRPS) [57], IFT81 (SRPS) [58], and IFT172 (SRPS) [59]. Like what we observed in our IFT74 cohort, the phenotypes in these patients ranged from BBS-like phenotypes to skeletal dysplasias with additional organ involvement. The phenotypes likely reflect the strength of the allele but also the particular subunit involved. For example, we now understand that some subunits like IFT74 are critical to IFT-B complex formation and so strongly deleterious alleles of these genes will affect ciliary assembly and give more severe phenotypes. Others like IFT25, IFT27, and IFT56 are not needed for ciliary assembly but play important roles in BBSome trafficking and Hedgehog signaling and are thus more likely to cause BBS like phenotypes [60–62].

Our studies combined with previously published studies of IFT74 patients present complicated but illuminating insight into the IFT74 structure function relationship. Loss of *IFT74* in mice leads to a severe phenotype with significant cardiac malformations. This is likely due to the failure of the IFT-B complex to assemble as IFT74 makes extensive contacts with other IFT-B proteins. Interestingly, we observed severe congenital heart defects in three of the five ATD/SRPS individuals reported here: double aortic arch in 2.II.2, persistent left superior vena cava, atrioventricular septal defect, hypoplastic left heart and aortic atresia in 3.II.1 and double outlet right ventricle and hypoplastic left ventricle in 4.II.1 No such heart defects have been previously reported in published BBS-, JBTS- or asthenozoospermia cases. The most severe human patients show SRPS/lethal ATD. These are often compound heterozygotes where one allele may be null due to a splicing defect early in the transcript and the other is a strong but not null allele. Splicing and other variants near the 3’end of the transcript result in BBS while a subtle variant near the N-terminus leads to isolated male infertility. Deletions near the N-terminus yield very different outcomes. In the mouse, replacement of the second coding exon with random residues produced no detectable phenotype indicating that it had little effect on the protein. In contrast removal of the first coding exon in either mouse or humans resulted in a complex phenotype combining ATD-/SRPS-like chondrodysplasia with features of mucociliary clearance / PCD disorders. While all published BBS- and JBTS cases as well as the two SRPS cases carrying splice variants exhibited polydactyly, this was not the case for the three ATD individuals identified here nor the previously published case carrying an exon 2 deletion. Our data suggests that this variant reduces tubulin binding but does not disrupt interaction with IFT81. The reduced tubulin binding likely causes axonemal microtubule extension deficits. The assembly defects result in shorter and fewer motile cilia that have a variety of structural abnormalities, all of which are likely to contribute to the reduced motility phenotype. The assembly defect appears to affect primary cilia to a milder degree, at least in mice. This difference is likely explained by higher demands for tubulin transport in motile cilia that are typically longer than primary cilia and may need more regeneration and repair due to mechanical stress not seen in primary cilia. Interestingly, the loss of Ift74 did not result in left-right asymmetry defects. This finding likely reflects our finding that primary cilia are less affected than motile cilia and the observation that very limited numbers of motile cilia are needed to break symmetry at the node [63].

## Materials and methods

### Ethics statement

Human subjects research was approved by the Ethics committee of the Institute of Child Health, University College London, London UK (GOSH R&D number 11MM03, REC number 08/H0713/82) the ethics committee Arnhem-Nijmegen, The Netherland (ethical approval no 2006–048) and the ethics committee of Freiburg University, Freiburg, Germany (votum no 122/20).

Mouse research was carried out at the University of Massachusetts Chan Medical School with Institutional Animal Care and Use Committee approval (PROTO201900265).

### Human subjects

This study involved individuals with the clinical diagnosis of ATD or SRPS. Diagnostic criteria included short ribs/narrow ribcage detected prenatally or postnatally with or without polydactyly and radiological signs such as trident acetabulum with spurs in pelvis x-rays in the first year of life, handlebar clavicles or cone shaped epiphyses after the first year of life). DNA samples were collected after obtaining written informed patient or parental consent.

### Mouse breeding

The *Ift74*^*Tm1a*^ mice were obtained from the KOMP project at Jackson Laboratory. These mice contain a genetrap insertion and selectable marker but have all exons intact. This allele was converted to the Tm1b allele by deleting the floxed exon with Stra8-iCre (stock 017490, Jackson Laboratory, Bar Harbor, ME, USA) [64], and to the Tm1c flox allele by deleting the genetrap insertion and selectable marker from the germline with C57Bl/6J congenic FlpE [65]. The flox Tm1c allele was converted to the Tm1d allele by deleting the floxed exon from Tm1c with Stra8-iCre. See Fig 4A for diagrams. All lines were C57Bl/6J congenics maintained by recurrent mating to wild type C57Bl/6J purchased from Jackson Laboratory (stock number 000664). Genotyping primers are provided in S3 Data.

### Statistics

Statistical results were obtained from at least three independent experiments. Statistical differences between groups were tested by t-tests, One-Way ANOVA, or Two-Way ANOVA as described in the figure legends. Differences between groups were considered statistically significant if *p* < 0.05. Otherwise, non-significant (n.s.) was labeled. Statistical significance is denoted with asterisks (* p < 0.05; ** p < 0.01; *** p < 0.001, **** p < 0.0001). Error bars indicate standard deviation (SD).

### Other procedures

Materials and Methods for other procedures are supplied in S1 Text.

## Supporting information

S1 Text Supplemental Experimental Procedures. (PDF)

S1 Table Summary of known *IFT74* variants. (PDF)

S2 Table Spreadsheet of Ift74Tm1a MicroCT phenotypes. (XLSX)

S3 Table Spreadsheet of Ift74Tm1a necropsy phenotypes. (XLSX)

S1 Fig Supplement to Support Fig 1. (A) IGV screenshot of exome data from showing absence of sequencing reads in the patient 1.II.1 while this exon is well covered in controls. Other exons in the patient are well covered indicating that only exon 2 is deleted in the patient. (B) IGV screenshot of *IFT74* exon 2 and surrounding intronic sequence from GS data in patient 1.II.1 showing the intronic breakpoints of the deletion. (C) Confirmation of the intronic breakpoints by Sanger sequencing in Family 1 showing the deletion of *IFT74* exon 2 as well as parts of intron 1 and depicting a small insertion (TTATTATACTC). The intronic breakpoints are at 5`g.26,959,921 and 3`g.26,962,978. (D) IGV screenshot of exome data from showing absence of sequencing reads in the patient 2.II.2 while this exon is well covered in controls. Other exons in the patient are well covered indicating that only exon 2 is deleted in the patient.
(TIF)

S2 Fig Minigene Assay. (A) Minigene analysis of how splicing is affected by the c.974+4>A variant. Insertion of the wild type or the c.974+4>A version of IFT74 exon 12 and surrounding intronic sequence between rhodopsin exons 3 and 5 resulted in product containing the rhodopsin exons spliced as expected to the IFT74 exon while mRNA from the mutant lacked the IFT74 exon. (B) Minigene analysis of how splicing is affected by the g.26982280delG variant. The wild type or the g.26982280delG version of IFT74 intron 4 including alternative exon 5 sequence was inserted between rhodopsin exons 3 and 5. Expression of this construct containing wild type sequence resulted in product containing rhodopsin exons 3 and 5 spliced with no IFT74 sequence included (w/o exon 5). The mutant form produced products with rhodopsin exons 3 and 5 spliced without exon 5 similar to wildtype and also produced a larger product with IFT74 alternative exon 5 (with exon 5) spliced between the two rhodopsin exons.
(TIF)

S3 Fig Supplement to Support Fig 9. (A-B) 3xHA-tagged human CBY, IFT46, and IFT52 were co-expressed in HEK293T cells with Flag-tagged IFT74 or IFT74Δexon2. Extracts were precipitated with HA beads and the eluants probed for Flag (A) or HA (B). Note that IFT46 and IFT52 brought down similar amounts of IFT74 and IFT74Δexon2 while no binding of wildtype or mutant IFT74 was observed with CBY. (C) In vitro translated wildtype IFT74, IFT74Δexon2, IFT74Δ79, or IFT74Δ120 was incubated with (+ tub) or without (- tub) microtubules and spun through a sucrose gradient. Note that microtubules pelleted (P) no wildtype and very little truncated IFT74 pelleted without tubulin. Full length IFT74 was enriched in the pellet (P) compared to the soluble (S) fraction, indicating tubulin binding while truncated IFT74 was enriched in the supernatant, indicating impaired tubulin binding. The ratios of the amount of IFT74 in the supernatant and pellet is indicated below the IFT74 western blot.
(TIF)

S4 Fig Supplement to Support Fig 9. (A) CP110 immunofluorescence analysis of cultured wildtype and IFT74 mutant MEFs reveals a slightly higher fraction of mutant cells with CP110 (green) present at both the mother and daughter centriole marked with gamma tubulin (red), ciliary axoneme marked with acetylated tubulin (red) (student t-test, p< 0.05), however over 90% of mutant cells showed no CP110 present at the mother centriole. (B) Immunofluorescence analysis of CBY (green) revealed presence at the ciliary base in both wildtype and IFT74 mutant cultured MEFs. Ciliary axoneme marked with ARL13B (red).
(TIF)

S1 Data Sequence of MmIft74 cDNA between exons 2 and 5. (PDF)

S2 Data Predicted Ift74 protein sequence in human and mouse alleles. (PDF)

S3 Data Genotyping Primers. (PDF)

S4 Data Supplemental to Support Fig 6: Ift74Tm1a Cilia. Quantification of ciliary defects by TEM (see Fig 6C).
(PDF)

S5 Data Supplemental to Support Fig 6: Ift74Tm1a Cilia. Localization of IFT proteins to MEF cilia (see Fig 6E).
(PDF)

S1 Video Live imaging of respiratory epithelia from 1.II.1 immediately after collection. (MOV)

S2 Video Live imaging of respiratory epithelia from 1.II.2- immediately after collection. (MOV)

S3 Video Live imaging of respiratory epithelia from 1.II.2 after explant was grown in culture for 6 weeks. (MOV)

S4 Video Live imaging of respiratory epithelia from a control patient immediately after collection. (MOV)
