# Supplementary material for: IFT74 variants cause skeletal ciliopathy and motile cilia defects in mice and humans
Source: PLoS Genet. 2023 Jun 14;19(6):e1010796. doi: 10.1371/journal.pgen.1010796 (PMC10298753; doi:10.1371/journal.pgen.1010796)
Supplement: S1 Text — (PDF) [file pgen.1010796.s001.pdf]

Supplemental Experimental Procedures

Supplemental Abbreviations

ATD, Asphyxiating Thoracic Dystrophy;

BBS, Bardet–Biedl syndrome;

DAPI, 4′,6-diamidino-2-phenylindole;

DMEM, Dulbecco's modified Eagle's medium;

E, embryonic day;

FBS, fetal bovine serum;

FCS, fetal calf serum;

JBTS, Joubert syndrome;

MEFs, Mouse embryonic fibroblasts;

P, postnatal day;

PCD, Primary Ciliary Dyskinesia;

SRPS, Short-Rib Polydactyly syndrome

Sequencing

Exome Sequencing

Exomic sequences from DNA samples were enriched with the SureSelect Human All Exon 50 V.6 Kit and 100-bp paired-end sequences were generated on a Hiseq PE150 (Illumina, San Diego, CA, USA). Read alignment and variant calling were performed with GATK using default parameters with the human genome assembly hg19 (GRCh37) as reference.

Following alignment and variant calling, serial variant filtering was performed as previously described (1, 2) for variants with a MAF equal or less than 0.5% in ExAc, 1000 genome project, esp6500 databases and gnom AD, coding variants or variants within 20 bp of exon-intron boundaries, and genes carrying bi-allelic variants with prioritization of homozygous variants in consanguineous pedigrees and genes with compound heterozygous variants in non-consanguineous pedigrees. Obligate loss of function variants such as canonical splice variants, frameshift and stop variants were prioritized over missense variants, however missense variants were not excluded from the analysis. BAM files were visually inspected for homozygous CNVs in all genes known to cause JATD or SRPS. CNV analysis was performed using ExomeDepth (3).

Genome sequencing and break point PCR

Genome sequencing from DNA sample of individual 1.II.1 was performed using the TruSeq DNA PCR-Free Kit (Illumina, San Diego, CA, USA) for library preparation which was sequenced as 2x150bp reads on a HiSeq4000 instrument (Illumina, San Diego, CA, USA). Read alignment and variant calling were performed using the GATK best practice pipeline with the human genome assembly hg19 (GRCh37) as reference. Reads were visualized using

the Integrative Genomics Viewer (IGV) to determine the breakpoints. Subsequently, breakpoint junctions were amplified with the primers F1: TCTGAAATGAGGGTCCAGCTA, R1: GGGTGTTTAAGAACTCTCCTCCT and F2: AGAAATGACAACTGGTAAAAACATT, R2: ATGACGAGTTAGTGGGTGCAG. Resultant fragments were separated by agarose gel electrophoresis and sequenced by Sanger dideoxy nucleotide sequencing.

Sanger sequencing

Genomic DNA was isolated by standard methods directly from blood samples by Qiagen kit (Qiagen, Hilden, Germany). Amplification of genomic DNA was performed in a volume of 50 µl containing 30 ng DNA, 50 pM of each primer, 2 mM dNTPs, and 1.0 U GoTaq DNA polymerase (#M3001, Promega, Madison, WI, USA). PCR amplifications were carried out by an initial denaturation step at 94°C for 3 min, and 33 cycles as follows: 94°C for 30 sec, 58-60°C for 30 sec, and 72°C for 70 sec, with a final extension at 72°C for 10 min. PCR products were verified by agarose gel electrophoresis, purified, and sequenced bi-directionally. Sequence data were evaluated using the CodonCode software. Primer sequences are available on request.

Splicing Assay

Minigene assays for the c.974+4 A>G and (IFT74-006 ENSB0000578614-g.26982286delG, E5) alleles were performed as previously described (4). In brief, amplified DNA fragments of 444 bp for c.974+4 A>G including *IFT74* exon 14 and 801 bp for (IFT74-006 ENSB0000578614-g.26982286-delg, E5) including *IFT74* exon 5 along with their flanking intronic sequences were cloned between *RHO* exon 3 and exon 5 in pCI-neo mammalian expression vector and the plasmid transfected into HEK293T cells. After 48 hours of incubation at 37 °C, total RNA was isolated by Qiagen kit (Qiagen, Hilden, Germany) and analyzed by reverse transcriptase PCR (iSCRIPT, BioRad, Hercules, CA, USA). *IFT74* Primer sequences are available on request. The amplified fragments were electrophoretically separated on agarose gel followed by purification and Sanger sequencing.

Microscopy (Schmidts Lab)

Transmission electron microscopy

Samples are fixed using 3% glutaraldehyde (EM-grade). After fixation, the samples were washed in distilled water, osmicated for in 1% OsO4 in water, and dehydrated in a series of ethanol steps (70%, 80%, 90%, 96%) via propylene oxide and embedded in LX-112 resin. After polymerization at 60°C, ultrathin sections of 60-70 nm were cut on a Reichert EM UC6 using a diamond knife and collected on copper grids and stained with uranyl acetate and lead citrate. Images of the sections were produced using a FEI Tecnai-12 electron microscope at 100 kVe (Fei, Eindhoven, The Netherlands), with a Veleta camera and Radius software (EMSIS, Münster, Germany).

High-speed videomicroscopy

High-speed videomicroscopy was performed as previously described (5) using a Leica DM IRB microscope coupled to a digital camera (Basler A602F-2) with frame grabber (Matrox meteor II/1394). Videos were taken at 200 frames per second.

Immunofluorescence Analysis

In brief, cells were grown on coverslips or slides prepared from nasal brushings (cells grown on coverslips were serum starved for 24 hrs) and washed with PBS before they were fixed in 4% PFA for 5 minutes, washed 3x with

PBS, permeabilized with PBS containing 0.05% Triton-X100, washed 3x in PBS, blocked in 5% BSA for 1 hour at room temperature or overnight at 4°C and then incubated overnight at 4°C in primary antibody 1:200 in 5% BSA in PBS. After washing for 5 times, secondary antibody solution was added (1:5000 in 5% BSA in PBS) and cells incubated for 2 hrs at room temperature. Slides and coverslips were mounted in Vectashield + 4′,6-diamidino-2-phenylindole (DAPI) (Vector Labs, Peterborough, UK). Images were taken with a Apotome Axiovert 200 (Zeiss, Oberkochen, Germany) and processed with AxioVision 4.8 (Zeiss, Oberkochen, Germany) or a confocal microscope (Olympus, Tokyo, Japan) and processed with Fiji (Imagej) software.

Antibodies

IFT74, rabbit polyclonal (HPA020247, Atlas antibodies, Bromma, Sweden),

CP110, rabbit polyclonal (12780-1-AP, Proteintech, Manchester, UK),

CBY, mouse monoclonal (8-2 sc-101551, Santa Cruz Biotechnology, Dallas, TX, USA),

ARL13B, rabbit polyclonal (17711-1-AP, Proteintech, Rosemont, IL, USA),

Acetylated-α-tubulin, mouse monoclonal (T7451, Sigma, Roedermark, Germany).

Anti-mouse Alexa Fluor 488 and anti-rabbit Alexa Fluor 546 were used as secondary antibodies (Molecular Probes Invitrogen, Carlsbad, CA USA).

Microscopy (Pazour Lab)

Hematoxylin and Eosin

Paraffin sections for H&E staining were dewaxed with Safeclear (Fisher Scientific, Hampton, NH) and rehydrated with graded aqueous solutions of isopropanol. The sections were stained for 4 min with CAT Hematoxylin (Biocare Medical, Concord, CA, USA), rinsed in running tap water for 30 sec followed by three quick dips in saturated lithium carbonate and a rinse in distilled water. This was followed by 90% ethanol for 2 min, Edgar Degas Eosin (Biocare Medical, Concord, CA, USA) for 2 min and 3 quick rinses in 100% ethanol. The sections were cleared with Safeclear (two 5 min incubations) and were mounted with Permount (Fisher Scientific, Hampton, NH, USA).

Immunofluorescence of Tissues

Paraffin sections were dewaxed, rehydrated and subjected to antigen retrieval in an autoclave (250°F, 40 min) with 10 mM sodium citrate at pH 6. Sections were brought to ambient temperature and treated with blocking solution (4% non-immune goat serum, 0.1% Triton X-100, 0.05% SDS, and 0.1% fish skin gelatin [Sigma] in TBST [0.05% Tween-20 in Tris-buffered saline, pH 7.4]) for 30 minutes, subsequently washed with TBST and then exposed to primary antibodies overnight at 4°C. Next day the sections were washed with TBST, incubated with Alexa Fluor-conjugated secondary antibodies (Life Technologies, Grand Island, NY, USA) for 30 min at 22°C, and washed with TBST followed by a rinse with TBS. The antibodies were brought to their working dilutions with 0.1% fish skin gelatin in TBS. The sections were then dipped for 5 seconds in DAPI (1 µg/ml in TBS) and after rinsing with TBS were mounted with Prolong Gold (Life Technologies, Carlsbad, CA, USA). Confocal images were acquired with an inverted microscope (TE-2000E2; Nikon, Tokyo, Japan) equipped with a Solamere Technology modified spinning disk confocal scan head (CSU10; Yokogawa, Tokyo, Japan). Z stacks were acquired at 0.5- micron intervals and

converted to single planes by maximum projection with MetaMorph software (MDS Analytical Technologies, Sunnyvale, CA, USA).

Electron Microscopy

Tracheas were fixed with 2.5% glutaraldehyde, 2% paraformaldehyde in 100 mM cacodylate pH 7.2 overnight before osmication with 1% osmium tetroxide for 1 hr at 22°C. For transmission EM, the bottom half of the trachea was dehydrated through the following series: 10%, 25% (plus 4% uranyl acetate), 30%, 50%, 70%, 85%, 95% and 100% ethanol and then infiltrated first with two changes of 100% propylene oxide and then with a 50%/50% propylene oxide/SPI-Pon 812 resin mixture for overnight incubation. The following day three changes of fresh 100% SPI-Pon 812 resin were done before the samples were polymerized at 68°C in plastic capsules. Thin sections of approximately 70 nm were placed on copper support grids and contrasted with lead citrate and uranyl acetate. Sections were examined using the FEI Tecnai (Thermo Fisher, Waltham, MA, USA) 12 BT with 80Kv accelerating voltage, and images were captured using a Gatan (Pleasanton, CA, USA) TEM CCD camera.

For scanning EM, the top half of the trachea was dehydrated through the following series: 10%, 30%, 50%, 70%, 85%, 95% and 100% ethanol and then critical point dried in liquid CO_2. The tracheas were cut in half longitudinally and mounted onto aluminum stubs with silver conductive paint and coated with carbon (1 nm) and sputter coated with gold/palladium (12 nm). Specimens were examined using an FEI (Hillsboro, OR, USA) Quanta 200 FEG MK II scanning electron microscope.

Immunofluorescence of Cells

Cells for immunofluorescence microscopy were grown on acid-washed glass coverslips. The cells were fixed for 10 min in -20° C methanol or for 15 min in 2% paraformaldehyde, 0.05 M Pipes, 0.025 M Hepes, 0.01 M EGTA, 0.01 M MgCl2 (pH 7.2) followed by a two-min extraction with 0.1% Triton X-100 in the same solution. For some antibodies, an antigen retrieval step of 0.05% SDS in PBS for 5 minutes included at this point. After two brief washes in TBST (0.01 M Tris, pH 7.5, 0.166 M NaCl, 0.05% Tween 20), the cells were blocked with 1% bovine serum albumin (BSA) in TBST for 1 hr and then incubated with the primary antibodies either overnight at 4°C or for 2 hr at room temperature. The cells were then washed 4 times with 1% BSA/TBST over ~30 min. The cells were then incubated with 1:2000 dilutions of Alexa fluor-conjugated secondary antibodies (Molecular Probes, Eugene, OR, USA) for 1 hr and washed 4 times with 1% BSA/TBST over ~30 min followed by a brief wash with TBST. The cells were then mounted with ProLong Antifade (Molecular Probes, Eugene, OR, USA) and visualized by fluorescence microscopy. Images were acquired by an Orca ER camera on a Zeiss Axiovert 200M microscope equipped with a Zeiss 100X plan-Apochromat 1.4 NA objective by Openlab (Lexington, MA, USA) and adjusted for contrast in Adobe Photoshop. If comparisons are to be made between images, the photos were taken with identical conditions and manipulated equally.

Quantification of fluorescence intensity was performed using an ImageJ macro-toolset (6) (https://imagej.net/ij/). Briefly, sets of images are imported into the software and cilia are pre-processed (median filtering and background subtraction), then detected using the “Analyze particle function”. Each cilium is extracted using a 64x64 pixels bounding box. Deconvolution is performed on both the structural marker channel and the

signal channel with the DeconvolutionLab plugin (7), using synthetic PSF generated by the Diffraction PSF 3D plugin (http://www.optinav.info/Diffraction-PSF-3D.htm).

Antibodies

Arl13b mouse monoclonal N295B/66 (Neuromab, University of California Davis, CA, USA),

Gamma tubulin mouse monoclonal GTU88 (Sigma, St. Louis, MO, USA),

Ift27 (8),

Ift140 (9),

Bbs9 polyclonal (Sigma, St. Louis, MO, USA, catalog number HPA021289),

Lztfl1 rabbit polyclonal (Proteintech, Rosemont, IL, USA),

Bbs3 rabbit polyclonal (10) (Gift of M. Nachury),

Anti-mouse and anti-rabbit Alexa Fluor (488, 594, 633) secondary antibodies (Molecular Probes Invitrogen, Carlsbad, CA USA).

Protein Analysis

MEF Western Blot

For western blots, MEFs were lysed directly into denaturing gel loading buffer (Tris-HCl 125mM pH6.8, Glycerol 20% v/v, SDS 4% v/v, β-Mercaptoethanol 10% v/v, bromophenol blue). Western blots were developed by chemiluminescence (Super Signal West Dura, Pierce Thermo, Waltham, MA,USA) and imaged using a LAS-3000 imaging system (Fujifilm, Tokyo, Japan).

Nasal Ciliated Cell Western Blot.

Nasal ciliated cell samples were obtained by gentle brushing deep inside the nasal cavities using a cervical brush (Frankmed, Germany). Cells were transferred from the brush to a 15 ml tube containing 5 ml of Dulbecco's modified Eagle's medium (DMEM) supplemented with 10% fetal calf serum (FCS), 1% pyruvate and 1% antibiotic mixture of penicillin and streptomycin and tubes centrifuged at 1200 rpm for 5 minutes to pellet the cells. Cell pellets were washed 3x with PBS and dry pellets snap frozen in liquid nitrogen.

Nasal epithelial cells were lysed in lysis buffer (50 mM Tris-HCl [pH 7.5], 150 mM NaCl, 1% NP-40) supplemented with complete protease inhibitor cocktail (Roche, Risch-Rotkreuz, Switzerland) and subjected to gel electrophoresis using a 4%–12% Bis-Tris SDS-PAGE gel (NuPAGE Novex, Thermo Fisher, Waltham, MA, USA) and blotted overnight. Blots were stained with anti-IFT74 antibody and fluorescence analyzed on a LI-COR Odyssey 2.1 (Thermo Fisher) infrared scanner.

Co-Immunoprecipitations.

Proteins tagged with 3xHA and 3xFLAG epitopes were generated from Gateway entry clones by LR Clonase reaction. All clones were confirmed by sequence analysis to match appropriate RefSeq genes. Overexpression of tagged versions of the proteins in HEK293T cells, whole-cell extracts and western blots were conducted as previously described (11). Plasmids expressing N-terminal 3x Flag-tagged IFT74 were co-transfected with plasmids expressing N-terminal 3x HA-tagged IFT81, IFT46, IFT52 and CBY in HEK293T cells. 24 hrs after transfection,

cells were lysed on ice using lysis buffer (50 mM Tris-HCl [pH 7.5], 150 mM NaCl, 1% NP-40) supplemented with complete protease inhibitor cocktail (Roche). Lysates were incubated with anti-HA affinity matrix (Roche) for 2–3 hr at 4°C. After incubation, beads with bound protein complexes were washed in ice-cold lysis buffer. Subsequently, 4X NuPAGE sample buffer was added to the beads and heated for 10 min at 70°C. Beads were pelleted by centrifugation, and supernatant was analyzed on NuPAGE Novex 4%–12% Bis-Tris SDS-PAGE gels. After blotting overnight at 4°C, blots were stained with mouse anti-FLAG or mouse anti-HA. Fluorescence was analyzed on a LI-COR Odyssey 2.1 infrared scanner and quantitated with ImageJ (6).

Tubulin binding assay.

The microtubule binding assay was performed according to the procedure provided by Cytoskeleton Inc. Briefly, 20ul of bovine microtubules (Cytoskeleton MT-001m Denver, CO, USA) and 150 ug of wildtype or mutant protein *in-vitro* translated using TnT® SP6 Quick Master Mix Coupled Transcription/Translation System (Promega, USA), according to the manufacturer`s instructions or recombinant wildtype or mutant protein purchased from Genscript (Piscataway, NJ, USA) (BacPower Guaranteed Bacterial Protein Expression, >90% purity) were incubated for 30min at RT. The samples were then layered on cushion buffer (Cytoskeleton, Denver, CO, USA) to optimize microtubule polymerization (Cytoskeleton, Denver, CO, USA) and centrifuged at 100,000 g for 40 minutes.

Cell culture

MEFs

Mutant MEFs were isolated from E13.5 mutant embryos and immortalized with the large T antigen from SV40 virus. MEFs and their derivatives were grown at 37°C in 5% CO_2 in Dulbecco’s modified Eagle’s medium (DMEM; Gibco Thermo Fisher) with 5% FBS and 1% Penicillin-Streptomycin (Gibco Thermo Fisher). All DNA constructs were transfected into the cells via lentiviral infection followed by drug selection to create stable cell lines.

Lentiviral Packaging

Lentiviral packaged pHAGE-derived plasmids (12) were packaged by a third generation system comprising four distinct packaging vectors (Tat, Rev, Gag/Pol, VSV-g) using HEK 293T cells as the host. DNA (Backbone: 5 μg; Tat: 0.5 μg; Rev: 0.5 μg; Gag/Pol: 0.5 μg; VSV-g: 1 μg) was delivered to the HEK cells using calcium phosphate precipitates. After 48 hours, supernatant was harvested, filtered through a 0.45 mm filter and precipitated with Lenti-X concentrator (Clontech). Precipitated viral particles were resuspended in growth medium and added to ~50% confluent cells. After 24 hrs, cells were selected with antibiotic.

Gli1 Expression

For SAG experiments, cells were plated at near confluent densities and serum starved (same culture medium described above but with 0.25% FBS) for 48 hours prior to treatment to allow ciliation. SAG (Calbiochem) was used at 400nM. Isolation of mRNA and quantitative mRNA analysis was performed as previously described (13) using these primers.

Primer	Accession No.	Sequence	Tm (C)	Amplicon
MmGAPDHExon3for	NM_008084	GCAATGCATCCTGCACCACCA	61.1	138 bp
MmGAPDHExon4rev		TTCCAGAGGGGCCATCCACA	61.1	

MmGli1Exon4for	NM_010296	CCAGGGTTATGGAGCAGCCAGA	61.2	135 bp
MmGli1Exon5rev		CTGGCATCAGAAAGGGGCGAGA	61.5	

Nasal Cell Culture

Nasal cell culture was performed as previously described (5, 14). In brief, nasal ciliated cells were obtained by gentle brushing deep inside the nasal cavities using a cervical brush (Frankmed, Germany. Cells were transferred from the brush to a 15 ml Falcon tube containing 5 ml of Dulbecco's modified Eagle's medium (DMEM) supplemented with 10% fetal calf serum, 1% pyruvate and 1% antibiotic mixture of penicillin and streptomycin. Cells were pelleted by centrifugation and treated with pronase over night before placement in a 25cm dish for 1 hr to remove fibroblasts. The supernatant was then transferred into a collagen coated T25 flask and cultured for three weeks. Upon confluency, cultures were treated with collagenase and cell sheets transferred into uncoated T25 flasks placed on a rotary shaker for one week followed by two weeks stationary incubation. Ciliated spheroids were kept in culture until used for imaging.

Plasmids

Plasmids were assembled using Gibson assembly (NEB, Ipswich, MA, USA) and the inserts and junctions verified by sequence. SnapGene files are available on request.

TE61 MmIft74 (MASNHK…ASRS*). Mouse cDNA was codon optimized and chemically synthesized (IDT, Skokie IL, USA). This was amplified and cloned in a pHAGE-derived vector (12) with an N-terminal 3X Flag tag. This construct is ampicillin resistant in bacteria and blasticidin resistant in mammalian cells.

GP726 MmIft74Δexon2 Met 2(MPPTT...ASRS*). The first coding exon (second exon of gene) from TE61 was deleted using a PCR approach with Gibson assembly. Initiation begins at the second methionine at the start of the second coding exon. The N-terminal Flag tag was maintained.

GP784 MmIft74 Met3 (MKTGMK...ASRS*). Nucleotides upstream from the third methionine were deleted from TE61 was deleted using a PCR approach with Gibson assembly. The N-terminal Flag tag was maintained.

GP785 MmIft74 Met4 (MKGPQR...ASRS*). Nucleotides upstream from the fourth methionine were deleted from TE61 was deleted using a PCR approach with Gibson assembly. The N-terminal Flag tag was maintained.

TE63 HsIft74 (MASNHK…TSGN*). The open reading frame of Hs*Ift74* was amplified from clone RC212777 (OriGene, Rockville MD, USA) and cloned into a pHAGE-derived vector with an N-terminal 3X Flag tag. This construct is ampicillin resistant in bacteria and blasticidin resistant in mammalian cells.

TE83 HsIft74Δexon2 (MPPGT...TSGN*). The open reading frame minus the first coding exon (second exon of gene) was amplified from TE63 and cloned into the same vector as TE63. The N-terminal Flag tag was maintained.

Supplemental References

1. Rehman AU, Najafi M, Kambouris M, Al-Gazali L, Makrythanasis P, Rad A, et al. Biallelic loss of function variants in PPP1R21 cause a neurodevelopmental syndrome with impaired endocytic function. Hum Mutat. 2019; 40 (3):267-80. pmid:30520571.

2. Schmidts M, Arts HH, Bongers EM, Yap Z, Oud MM, Antony D, et al. Exome sequencing identifies DYNC2H1 mutations as a common cause of asphyxiating thoracic dystrophy (Jeune syndrome) without major polydactyly, renal or retinal involvement. J Med Genet. 2013; 50 (5):309-23. pmid:23456818.

3. Plagnol V, Curtis J, Epstein M, Mok KY, Stebbings E, Grigoriadou S, et al. A robust model for read count data in exome sequencing experiments and implications for copy number variant calling. Bioinformatics. 2012; 28 (21):2747-54. pmid:22942019.

4. Maria M, Lamers IJ, Schmidts M, Ajmal M, Jaffar S, Ullah E, et al. Genetic and clinical characterization of Pakistani families with Bardet-Biedl syndrome extends the genetic and phenotypic spectrum. Sci Rep. 2016; 6:34764. pmid:27708425.

5. Paff T, Loges NT, Aprea I, Wu K, Bakey Z, Haarman EG, et al. Mutations in PIH1D3 Cause X-Linked Primary Ciliary Dyskinesia with Outer and Inner Dynein Arm Defects. American journal of human genetics. 2017; 100 (1):160-8. pmid:28041644.

6. Schneider CA, Rasband WS, Eliceiri KW. NIH Image to ImageJ: 25 years of image analysis. Nat Methods. 2012; 9 (7):671-5. pmid:22930834.

7. Sage D, Donati L, Soulez F, Fortun D, Schmit G, Seitz A, et al. DeconvolutionLab2: An open-source software for deconvolution microscopy. Methods. 2017; 115:28-41. pmid:28057586.

8. Keady BT, Samtani R, Tobita K, Tsuchya M, San Agustin JT, Follit JA, et al. IFT25 links the signal-dependent movement of Hedgehog components to intraflagellar transport. Dev Cell. 2012; 22 (5):940-51. pmid:22595669.

9. Jonassen JA, SanAgustin J, Baker SP, Pazour GJ. Disruption of IFT complex A causes cystic kidneys without mitotic spindle misorientation. J Am Soc Nephrol. 2012; 23 (4):641-51. pmid:22282595.

10. Jin H, White SR, Shida T, Schulz S, Aguiar M, Gygi SP, et al. The conserved Bardet-Biedl syndrome proteins assemble a coat that traffics membrane proteins to cilia. Cell. 2010; 141 (7):1208-19. pmid:20603001.

11. Loges NT, Antony D, Maver A, Deardorff MA, Gulec EY, Gezdirici A, et al. Recessive DNAH9 Loss-of-Function Mutations Cause Laterality Defects and Subtle Respiratory Ciliary-Beating Defects. American journal of human genetics. 2018; 103 (6):995-1008. pmid:30471718.

12. Wilson AA, Kwok LW, Hovav AH, Ohle SJ, Little FF, Fine A, et al. Sustained expression of alpha1-antitrypsin after transplantation of manipulated hematopoietic stem cells. Am J Respir Cell Mol Biol. 2008; 39 (2):133-41. pmid:18323534.

13. Jonassen JA, San Agustin J, Follit JA, Pazour GJ. Deletion of IFT20 in the mouse kidney causes misorientation of the mitotic spindle and cystic kidney disease. J Cell Biol. 2008; 183 (3):377-84. pmid:18981227.

14. Willems T, Jorissen M. Sequential monolayer-suspension culture of human airway epithelial cells. J Cyst Fibros. 2004; 3 Suppl 2:53-4. pmid:15463926.
