# Supplementary material for: IFT74 variants cause skeletal ciliopathy and motile cilia defects in mice and humans
Source: PLoS Genet. 2023 Jun 14;19(6):e1010796. doi: 10.1371/journal.pgen.1010796 (PMC10298753; doi:10.1371/journal.pgen.1010796)
Supplement: S1 Table — (PDF) [file pgen.1010796.s002.pdf]

Supplemental Table 1: Summary of known *IFT74* variants

**ATD/ PCD/ SRPS**	Authors	Bakey et al. Pazour, 2023	Bakey et al. Pazour, 2023	Bakey et al. Pazour, 2023	Bakey et al. Pazour, 2023	Bakey et al. Pazour, 2023	Hammarsjö et al. Grigelioniene
	Identifier	1.II.1	1.II.2	2.II.2	3.II.1	4.II.1	Family 20
	Reference	This work	This work	This work	This work	This work	PMID: 33875766
	Disease	ATD / PCD	ATD / PCD	ATD	ATD / SRPS	ATD / SRPS	ATD / SRPS
	IFT74 Variant 1	Exon 2 deletion (3kb) g.26959922_26962977delinsTTATTATACTC	Exon 2 deletion (3kb) g.26959922_26962977delinsTTATTATACTC	Exon 2 deletion (3kb) g.26959922_26962977delinsTTATTATACTC	c.974+4A>G (splice donor, intron 12)	c.789+2T>G (splice donor, intron 10)	Exon 2 deletion (3kb) g.26959922_26962969delinsTTA
	IFT74 Variant 2	Exon 2 deletion (3kb) g.26959922_26962977delinsTTATTATACTC	Exon 2 deletion (3kb) g.26959922_26962977delinsTTATTATACTC	Exon 2 deletion (3kb) g.26959922_26962977delinsTTATTATACTC	c.305+1664delG (splice donor, intron 4)	c.789+2T>G (splice donor, intron 10)	Exon 2 deletion (3kb) g.26959922_26962969delinsTTA
**JBTS**	Authors	Luo et al. Cao 2021	Luo et al. Cao 2021	Luo et al. Cao 2021	Luo et al. Cao 2021	Zhongling et al. Xiaoru 2021	
	Identifier	78C1 (Family 1_II:1)	78C2 (Family 1_II:2)	103C (Family 2_II:1)	117C (Family 3_II:1)		
	Reference	PMID: 33531668	PMID: 33531668	PMID: 33531668	PMID: 33531668	PMID: 34539760	
	Disease	JBTS	JBTS	JBTS	JBTS	JBTS	
	IFT74 Variant 1	c.92delT (p.Leu31Hfs*25)	c.92delT (p.Leu31Hfs*25)	c.306-24A>G [#] (p.Ser103_Arg135del)	c.85C>T (p.Arg29*)	c.853G>T (p.E285*)	
	IFT74 Variant 2	c.535C>G (p.Q179E)	c.535C>G (p.Q179E)	c.535C>G (p.Q179E)	c.535C>G (p.Q179E)	c.535C>G (p.Q179E)	
				[#]causes skipping of exon 5 resulting in an in-frame 33-amino acid deletion from Ser103 to Arg135			
**BBS**	Authors	Lindstrand et al. Katsanis 2016	Kleinendorstvan et al. Haelst 2020	Mardy et al. Slavotinek 2021			
	Identifier						
	Reference	PMID: 27486776	PMID: 32144365	PMID: 33748949			
	Disease	BBS	BBS	BBS			
	IFT74 Variant 1	Deletion of exon 14–19 (Exon 14 starts with Gly352; exon 19 ends with Glu561)	c.371_372del (p.Q124Rfs*9)	c.1685-1G>T [#] (splice defect, intron 19)			
	IFT74 Variant 2	c.1685-1G>T [#] (splice defect, intron 19)	c.1685-1G>T [#] (splice defect, intron 19)	c.1685-1G>T [#] (splice defect, intron 19)			
		[#]Exon 20 starts at Phe562. This variant could delete the last 39 residues.					
**Male Infer-tility**	Authors	Lorès et al. Touré 2021	Lorès et al. Touré 2021				
	Identifier	patient 1	patient 2				
	Reference	PMID: 33689014	PMID: 33689014				
	Disease	Spermatogenic failure	Spermatogenic failure				
	IFT74 Variant 1	c.256G > A [#] (p.Gly86Ser) and (p.Leu77_Gly86del)	c.256G > A [#] (p.Gly86Ser) and (p.Leu77_Gly86del)				
	IFT74 Variant 2	c.256G > A [#] (p.Gly86Ser) and (p.Leu77_Gly86del)	c.256G > A [#] (p.Gly86Ser) and (p.Leu77_Gly86del)				
		[#]Causes a splicing defect that produces aberrant messages. A common variant deletes amino acids Leu77 through Gly86					
