# Supplementary figures and images for: IFT74 variants cause skeletal ciliopathy and motile cilia defects in mice and humans
Source: PLoS Genet. 2023 Jun 14;19(6):e1010796. doi: 10.1371/journal.pgen.1010796 (PMC10298753; doi:10.1371/journal.pgen.1010796)

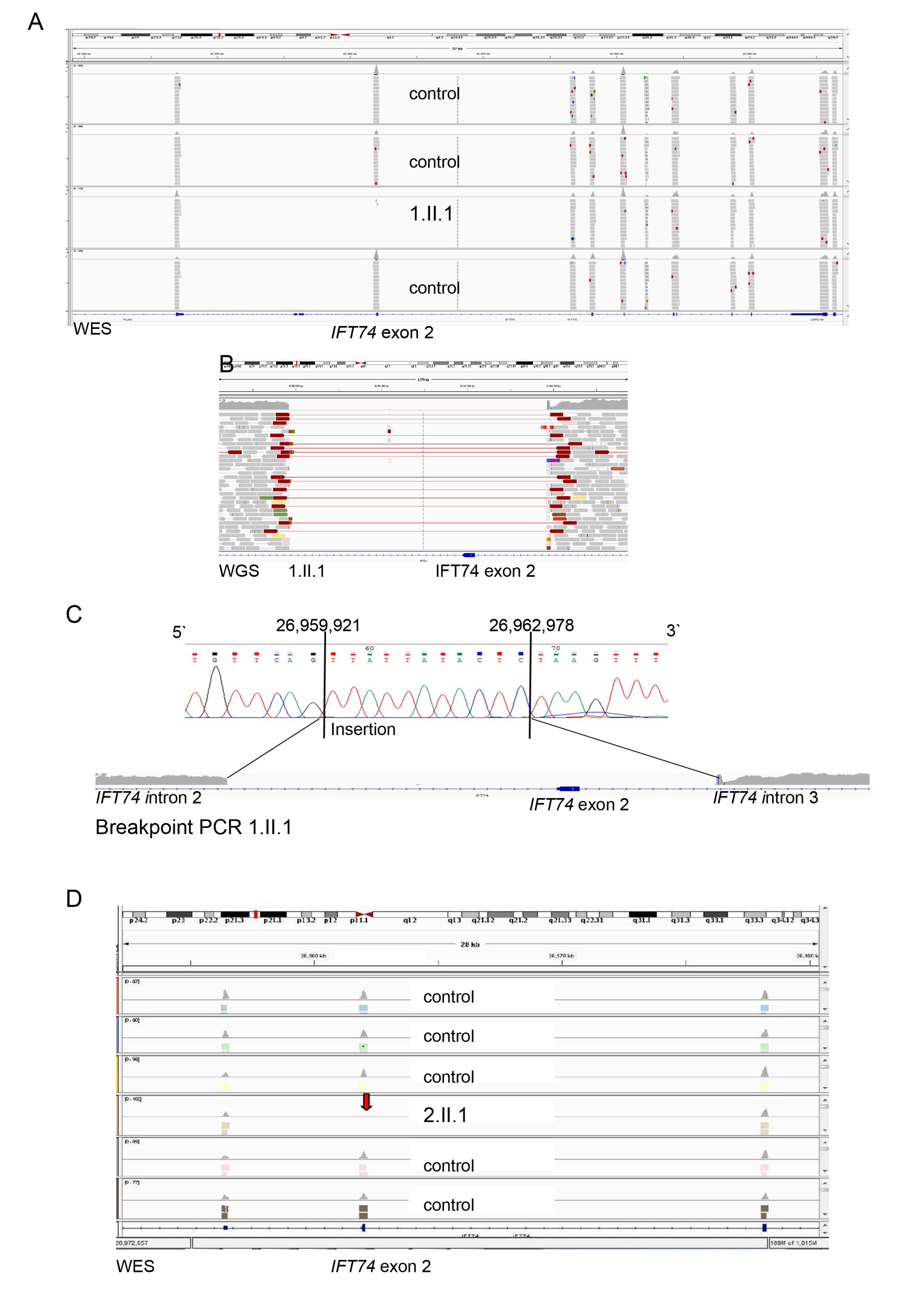

Supplement: S1 Fig — (A) IGV screenshot of exome data from showing absence of sequencing reads in the patient 1.II.1 while this exon is well covered in controls. Other exons in the patient are well covered indicating that only exon 2 is deleted in the patient. (B) IGV screenshot of IFT74 exon 2 and surrounding intronic sequence from GS data in patient 1.II.1 showing the intronic breakpoints of the deletion. (C) Confirmation of the intronic breakpoints by Sanger sequencing in Family 1 showing the deletion of IFT74 exon 2 as well as parts of intron 1 and depicting a small insertion (TTATTATACTC). The intronic breakpoints are at 5`g.26,959,921 and 3`g.26,962,978. (D) IGV screenshot of exome data from showing absence of sequencing reads in the patient 2.II.2 while this exon is well covered in controls. Other exons in the patient are well covered indicating that only exon 2 is deleted in the patient. (TIF) [file pgen.1010796.s005.tif]

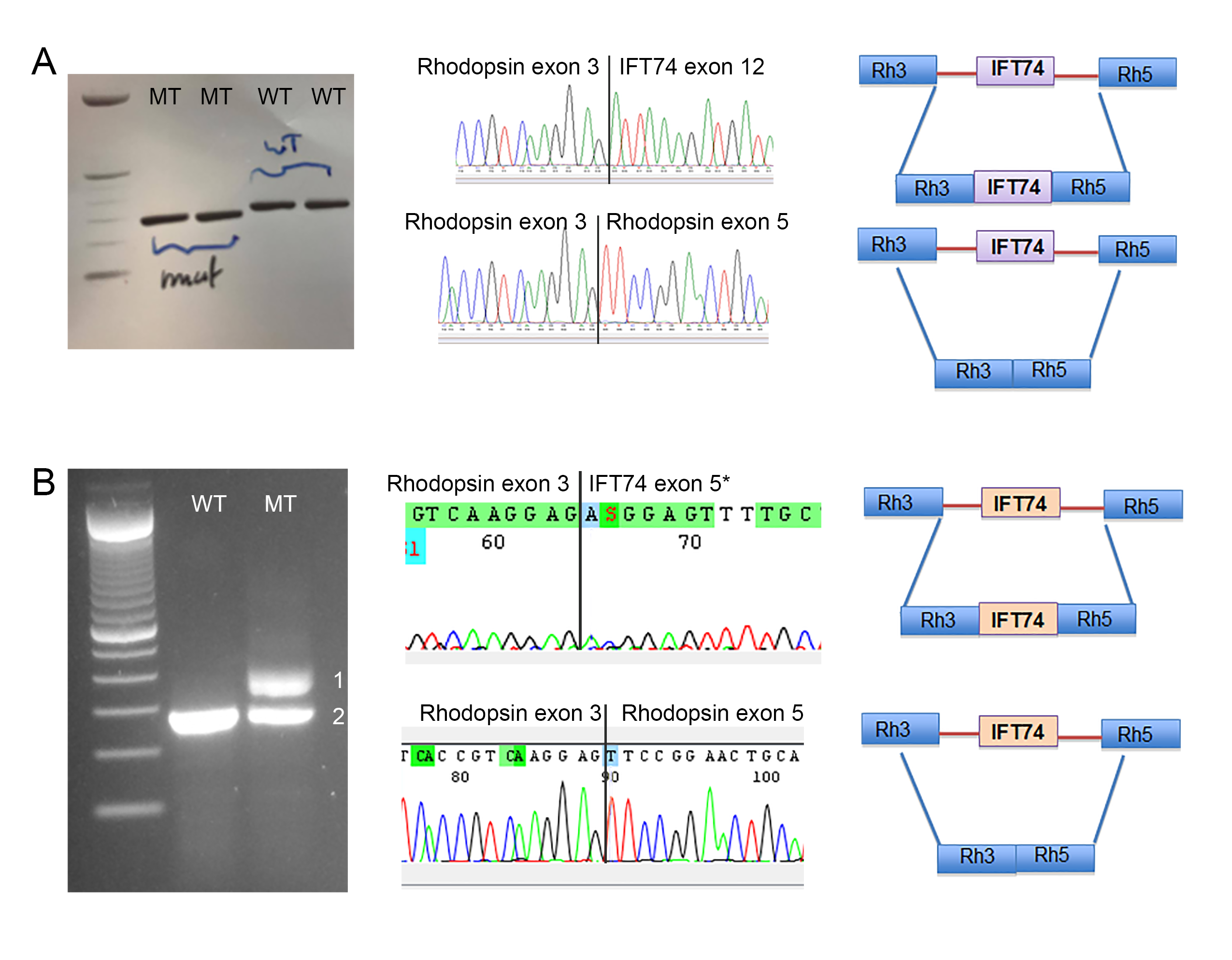

Supplement: S2 Fig — (A) Minigene analysis of how splicing is affected by the c.974+4>A variant. Insertion of the wild type or the c.974+4>A version of IFT74 exon 12 and surrounding intronic sequence between rhodopsin exons 3 and 5 resulted in product containing the rhodopsin exons spliced as expected to the IFT74 exon while mRNA from the mutant lacked the IFT74 exon. (B) Minigene analysis of how splicing is affected by the g.26982280delG variant. The wild type or the g.26982280delG version of IFT74 intron 4 including alternative exon 5 sequence was inserted between rhodopsin exons 3 and 5. Expression of this construct containing wild type sequence resulted in product containing rhodopsin exons 3 and 5 spliced with no IFT74 sequence included (w/o exon 5). The mutant form produced products with rhodopsin exons 3 and 5 spliced without exon 5 similar to wildtype and also produced a larger product with IFT74 alternative exon 5 (with exon 5) spliced between the two rhodopsin exons. (TIF) [file pgen.1010796.s006.tif]

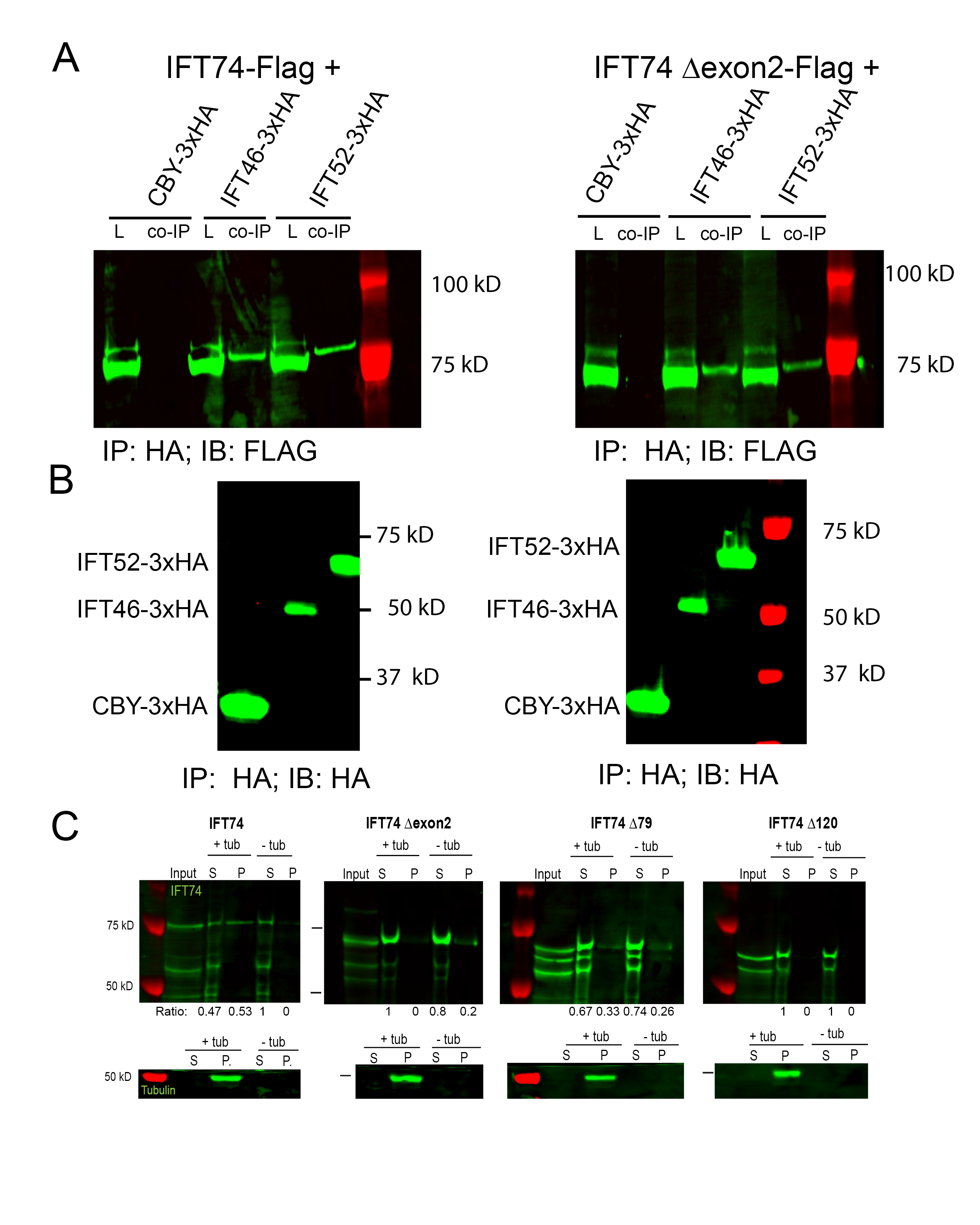

Supplement: S3 Fig — (A-B) 3xHA-tagged human CBY, IFT46, and IFT52 were co-expressed in HEK293T cells with Flag-tagged IFT74 or IFT74Δexon2. Extracts were precipitated with HA beads and the eluants probed for Flag (A) or HA (B). Note that IFT46 and IFT52 brought down similar amounts of IFT74 and IFT74Δexon2 while no binding of wildtype or mutant IFT74 was observed with CBY. (C) In vitro translated wildtype IFT74, IFT74Δexon2, IFT74Δ79, or IFT74Δ120 was incubated with (+ tub) or without (- tub) microtubules and spun through a sucrose gradient. Note that microtubules pelleted (P) no wildtype and very little truncated IFT74 pelleted without tubulin. Full length IFT74 was enriched in the pellet (P) compared to the soluble (S) fraction, indicating tubulin binding while truncated IFT74 was enriched in the supernatant, indicating impaired tubulin binding. The ratios of the amount of IFT74 in the supernatant and pellet is indicated below the IFT74 western blot. (TIF) [file pgen.1010796.s007.tif]

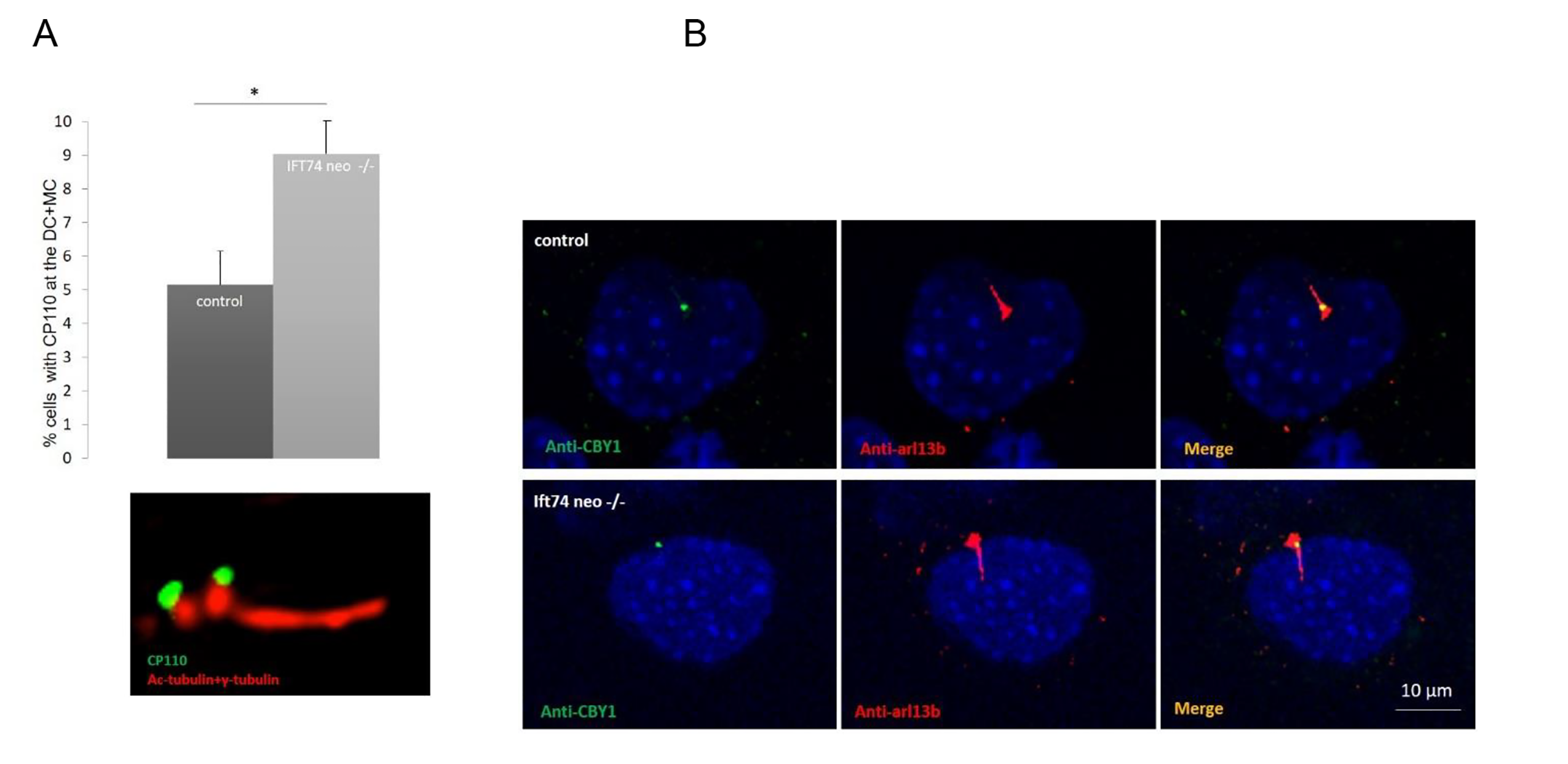

Supplement: S4 Fig — (A) CP110 immunofluorescence analysis of cultured wildtype and IFT74 mutant MEFs reveals a slightly higher fraction of mutant cells with CP110 (green) present at both the mother and daughter centriole marked with gamma tubulin (red), ciliary axoneme marked with acetylated tubulin (red) (student t-test, p< 0.05), however over 90% of mutant cells showed no CP110 present at the mother centriole. (B) Immunofluorescence analysis of CBY (green) revealed presence at the ciliary base in both wildtype and IFT74 mutant cultured MEFs. Ciliary axoneme marked with ARL13B (red). (TIF) [file pgen.1010796.s008.tif]
