# Supplementary material for: IFT74 variants cause skeletal ciliopathy and motile cilia defects in mice and humans
Source: PLoS Genet. 2023 Jun 14;19(6):e1010796. doi: 10.1371/journal.pgen.1010796 (PMC10298753; doi:10.1371/journal.pgen.1010796)
Supplement: S1 Data — (PDF) [file pgen.1010796.s009.pdf]

Supplemental Data 1: Sequence of MmIft74 cDNA between exons 2 and 5.

Primers:	MmIft74_exon2_for	ctcggggtggaataggactaacagg
	MmIft74_exon5_rev	gaagcttattaatttcagttgtaagttcac

>MmIft74$^{\text{wild type}}$
Ctcggggtggaataggactaacaggaaggcctccttctggaataagacctccatctggcaatgttcgagtggcaactgcaatgccaccaacaacagcaagaccaggtt
ctcgtggtggtcccttagggactggtggagttttgtcatctcaaatcaaagttgctgatcgtcctgtgacccaacaaggtttgagtggaatgaagactggcatgaaaggtcc
ccagaggcaaattttagacaaatcttactatcttggacttcttaggagcaaaataagtgaacttacaactgaaattaataagcttc

>MmIft74$^{\text{Tm1a}}$
ctcggggtggaataggactaacaggaaggcctccttctggaataagacctccatctggcaatgttcgagtggcaactgcaGTCCCAGGTCCCGAAAAC
CAAAGAAGAAGAACCCTAACAAAGAGGACAAGCGGCCTCGCACAGCCTTCACTGCTGAGCAGCTCCA
GAGGCTCAAGGCTGAGTTTCAGACCAACAGatgccaccaacaacagcaagaccaggttctcgtggtggtcccttagggactggtggagtttt
gtcatctcaaatcaaagttgctgatcgtcctgtgacccaacaaggtttgagtggaatgaagactggcatgaaaggtccccagaggcaaattttagacaaatcttactatcttg
gacttcttaggagcaaaataagtgaacttacaactgaaattaataagcttc

>MmIft74$^{\text{Tm1b}}$
ctcggggtggaataggactaacaggaaggcctccttctggaataagacctccatctggcaatgttcgagtggcaactgcagtcccaGGTCCCGAAAACCA
AAGAAGAAGAACCCTAACAAAGAGGACAAGCGGCCTCGCACAGCCTTCACTGCTGAGCAGCTCCAGA
GGCTCAAGGCTGAGTTTCAGACCAACaggtccccagaggcaaattttagacaaatcttactatcttggacttcttaggagcaaaataagtgaactta
caactgaaattaataagcttc

>MmIft74$^{\text{Tm1d}}$
ctcggggtggaataggactaacaggaaggcctccttctggaataagacctccatctggcaatgttcgagtggcaactgcagtccccagaggcaaattttagacaaatctta
ctatcttggacttcttaggagcaaaataagtgaacttacaactgaaattaataagcttc
