# Supplementary material for: IFT74 variants cause skeletal ciliopathy and motile cilia defects in mice and humans
Source: PLoS Genet. 2023 Jun 14;19(6):e1010796. doi: 10.1371/journal.pgen.1010796 (PMC10298753; doi:10.1371/journal.pgen.1010796)
Supplement: S2 Data — (PDF) [file pgen.1010796.s010.pdf]

Supplemental Data 2: Predicted Ift74 protein sequence in human and mouse alleles.

>HsIFT74 Wild Type
MASNHKSSAPRPISRGGIGLTGRPPSGIRPPSGNVRVATAMPPTTARPGSRGGPLGTGGVLSSQIKVAHRPVTQQGLTGMKTGTKGPQRQILDKSYYLGLLRSKISELTTEVN…
>HsIFT74 Predicted deletion resulting from loss of exon 2
MPPTTARPGSRGGPLGTGGVLSSQIKVAHRPVTQQGLTGMKTGTKGPQRQILDKSYYLGLLRSKISELTTEVN…

>MmIft74 Wild type
MASNHKSSAPRPISRGGIGLTGRPPSGIRPPSGNVRVATAMPPTTARPGSRGGPLGTGGVLSSQIKVADRPVTQQGLSGMKTGMKGPQRQILDKSYYLGLLRSKISELTTEIN…
>MmIft74 Tm1a
MASNHKSSAPRPISRGGIGLTGRPPSGIRPPSGNVRVATAVPGPENQRRRTLTKRTSGLAQPSLLSSSRGSRLSFRPTDATNNSKTRFSWWSLRDWWSFVISNQSC*
>MmIft74 Tm1a Reinitiation
MPPTTARPGSRGGPLGTGGVLSSQIKVADRPVTQQGLSGMKTGMKGPQRQILDKSYYLGLLRSKISELTTEIN…
>MmIft74 Tm1b
MASNHKSSAPRPISRGGIGLTGRPPSGIRPPSGNVRVATAVPGPENQRRRTLTKRTSGLAQPSLLSSSRGSRLSFRPT-------GPQRQILDKSYYLGLLRSKISELTTEIN…
>MmIft74 Tm1d
MASNHKSSAPRPISRGGIGLTGRPPSGIRPPSGNVRVATAVPRGKF*
GPQRQILDKSYYLGLLRSKISELTTEIN…

Methionines are in red text
Exon 3 is underlined
Non-native Ift74 sequence
