# Supplementary material for: IFT74 variants cause skeletal ciliopathy and motile cilia defects in mice and humans
Source: PLoS Genet. 2023 Jun 14;19(6):e1010796. doi: 10.1371/journal.pgen.1010796 (PMC10298753; doi:10.1371/journal.pgen.1010796)
Supplement: S3 Data — (PDF) [file pgen.1010796.s011.pdf]

Supplemental Data 3: Genotyping Primers

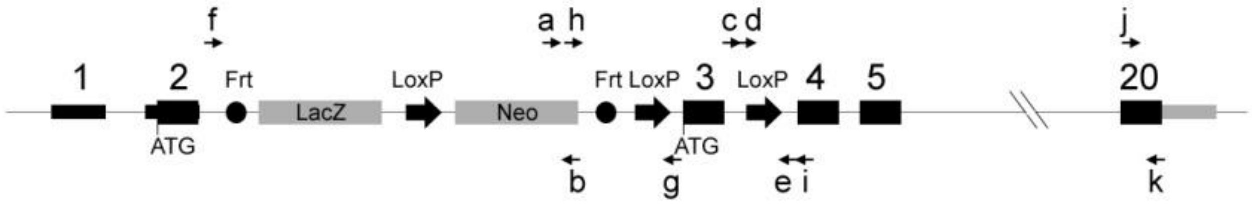

a: LacZ Fwd	TACCGTTGATGTTGAAGTGGC
b: LacZ Rev	GACTGTAGCGGCTGATGTTG
c: IFT74_WT_Fwd.1	GAATGCATGTGAAATACATTGTGAA
d: IFT74_Intron3_loxP_fwd.1	CGCAATTAATGATAACTTCGTATAGC
e: IFT74_intron3_Rev	GAGAAAAGCAGTAATAGTTCTCATCTCC
f: IFT74_intron2_Frt_Fwd.2	CTGAGTGAAAGTGGAGGC
g: IFT74_intron2_Frt2_Rev.3	CAAGAAAGCTGGGTCTAGAT
h: IFT74_LacZ_SV40_Fwd2	TAATAATAACCGGGCAGGGG
i: IFT74_Intron3_Rev.1	GAGGGAAGCATAATGTCAGTC
j: 796_Ift74_F	CCTCACTTTATTTMAGAATGCAAGC
k:796_Ift74_R	GTTACAGAACAGAAACTGGTGCT

a+b = LacZ band of approximately 300bp

c+e = Wild type band of approximately 400bp

d+e = Tm1a band of approximately 400bp

f+g = Tm1c band of approximately 200bp and wild type band of approximately 300bp

h+i = Tm1b band of approximately 700bp

e+f = Tm1e band of 539 bp and 1298 bp in wild type

j+k = 300 bp band that cuts in half with AciI when the *Ift74*^{b2b796Clo} mutation is present
