# Supplementary material for: IFT74 variants cause skeletal ciliopathy and motile cilia defects in mice and humans
Source: PLoS Genet. 2023 Jun 14;19(6):e1010796. doi: 10.1371/journal.pgen.1010796 (PMC10298753; doi:10.1371/journal.pgen.1010796)
Supplement: S4 Data — Quantification of ciliary defects by TEM (see Fig 6C). (PDF) [file pgen.1010796.s012.pdf]

Supplemental Data 4. Supplemental to Support Figure 6: Ift74^{Tm1a} Cilia.

Quantification of ciliary defects by TEM (see Figure 6C)

Cilia from control and *Ift74^{Tm1a}* mutant trachea were imaged by TEM and ciliary defects counted.

Animal	Age	*Ift74^{Tm1a}* Genotype	Missing Central Pair	Super-numary MTs	Missing MTs	Displaced Outer Doublets	Unorganized Cilia	Normal	Total	Percentage of defective cilia
21297	P19	Mut	1	4	2	1	2	110	120	8.3
21298	P19	Mut	0	0	0	0	1	109	110	0.9
21300	P19	Het	0	0	0	0	0	62	62	0
21301	P19	Het	0	0	0	0	1	70	71	1.4
21798	P15	Mut	0	14	12	3	9	189	227	16.7
21800	P15	WT	0	2	0	0	2	274	278	1.4
21828	P8	Het	0	0	0	0	0	147	147	0
21829	P8	Mut	2	8	11	3	1	203	228	11.0
