# Supplementary material for: IFT74 variants cause skeletal ciliopathy and motile cilia defects in mice and humans
Source: PLoS Genet. 2023 Jun 14;19(6):e1010796. doi: 10.1371/journal.pgen.1010796 (PMC10298753; doi:10.1371/journal.pgen.1010796)
Supplement: S5 Data — Localization of IFT proteins to MEF cilia (see Fig 6E). (PDF) [file pgen.1010796.s013.pdf]

Supplemental Data 5. Supplemental to Support Figure 6: Ift74^{Tm1a} Cilia.

Localization of IFT proteins to MEF cilia (see Figure 6E)

Localization of IFT proteins in wild type and Ift74^{Tm1a} MEF cilia. N=3 cell lines for each genotype, 25 cells examined from each cell line. P determined by student t-test.

IFT27	WT 100+/-0% spot at the base<br>MT 39+/-10% spot at the base<br>P=0.0005
IFT140	WT 100+/-0% spot at the base<br>MT 100+/-0% spot at the base<br>P=ns
BBS9	WT 57+/-10% spot at the base<br>MT 74+/-8% spot at the base<br>P=ns
Lztfl1	WT 100+/-0% no cilia label<br>MT 100+/-0% no cilia label<br>P=ns
BBS3	WT 100+/-0% no cilia label<br>MT 100+/-0% no cilia label<br>P=ns
